# Real-world uptake of nirsevimab, RSV maternal vaccine, and RSV vaccines for older adults: a systematic review and meta-analysis

**DOI:** 10.1016/j.eclinm.2025.103281

**Published:** 2025-06-03

**Authors:** Daira Trusinska, Bohee Lee, Sohail Ferdous, Harley H.Y. Kwok, Becky Gordon, Junyi Gao, Liantao Ma, Hanbiao Xiong, Sir Aziz Sheikh, Jürgen Schwarze, John Busby, Cheryl Gibbons, Simon B. Drysdale, Sir Lewis Ritchie, Thomas Williams, Ting Shi

**Affiliations:** a Usher Institute, Edinburgh Medical School, University of Edinburgh, Edinburgh, UK; b National Heart & Lung Institute, Imperial College London, London, UK; c School of Public Health, Imperial College London, London, UK; d National Engineering Research Center for Software Engineering, Peking University, Beijing, China; e Nuffield Department of Primary Care Health Sciences, University of Oxford, Oxford, UK; f Child Life and Health, Centre for Inflammation Research, Institute for Regeneration and Repair, The University of Edinburgh, UK; g Centre for Public Health, Queen's University Belfast School of Medicine Dentistry and Biomedical Sciences, Belfast, UK; h Vaccination and Immunisation Division, Clinical and Protecting Health Directorate, Public Health Scotland, UK; i Oxford Vaccine Group, Department of Paediatrics, University of Oxford, Oxford, UK; j The NIHR Oxford Biomedical Research Centre, Oxford, UK; k Academic Primary Care, University of Aberdeen, UK

**Keywords:** Respiratory syncytial virus (RSV), Immunisation programmes, Vaccine uptake, Nirsevimab, RSV vaccines

## Abstract

**Background:**

In clinical trials, recently introduced respiratory syncytial virus (RSV) immunisation products have shown high efficacy in preventing severe RSV outcomes. Implementing successful immunisation programmes is however key to realising these benefits in real-world settings. We aimed to investigate uptake of the long-acting monoclonal antibody nirsevimab, the RSV maternal vaccine, and RSV vaccines for older adults in countries where immunisation programmes have been introduced, and to explore how uptake varies between countries and population subgroups.

**Methods:**

In this systematic review and meta-analysis, we carried out four monthly searches in Medline, Embase, and Global Health databases for studies reporting uptake of nirsevimab, the RSV maternal vaccine, and RSV vaccines for older adults. We included population-based studies published between December 1, 2022 and February 5, 2025. Two independent reviewers screened studies, extracted data, and completed a risk of bias assessment using the Joanna Briggs Institute (JBI) Critical Appraisal Tools. We assessed uptake stratified by country and socio-demographic and clinical subgroups. Meta-analyses were conducted using random-effects modelling. PROSPERO registration number: CRD42025643585.

**Findings:**

We screened a total of 1267 studies, 43 of which met the inclusion criteria reporting data on over 1.38 million individuals from six countries. Nirsevimab uptake data were reported in 34 studies: 16 from Spain, eight from the United States, seven from France, one with combined data from Andorra and Spain, and one from each of Italy and Luxembourg. Our pooled estimates showed that nirsevimab uptake on population level was 90.1% (95% confidence interval (CI): 86.4–92.9) in Spain and 51.2% (95% CI: 29.3–72.7) in the United States during the 2023/24 RSV season. Uptake data for the RSV maternal vaccine and RSV vaccines for older adults were reported in five and eight studies, respectively, all from the United States. Meta-analysis showed population-level uptake of 30.5% (95% CI: 20.6–42.6) and 18.2% (95% CI: 10.8–28.9), respectively. Uptake varied across subgroups.

**Interpretation:**

Uptake of nirsevimab varied substantially between the countries that have implemented infant RSV immunisation programmes. Despite the limited number of studies and the lack of more accurate data at national level the low uptake estimates for RSV maternal vaccine and RSV vaccines for older adults are concerning. National, clinical, and public health initiatives are needed to increase uptake of RSV immunisation products and ensure maximum benefit to people currently at risk of severe RSV outcomes.

**Funding:**

10.13039/501100023699Health Data Research UK, Inflammation and Immunity Driver Programme.

**Research in context**

**Evidence before this study**

Globally, respiratory syncytial virus (RSV) is the leading cause of acute respiratory infections in infants under 12 months of age, as well as a substantial threat for older adults. Recently, new prophylactic options have been approved for use in Europe, the United Kingdom, and North America: long-acting monoclonal antibody nirsevimab, RSV vaccine for pregnant individuals, and RSV vaccines for older adults. As these immunisation products are introduced, it is crucial to monitor and evaluate their uptake in real-world settings. To our knowledge, this is the first systematic review and meta-analysis synthesising evidence on uptake of RSV immunisation products worldwide.

**Added value of this study**

RSV uptake data on nirsevimab, RSV maternal vaccine and vaccines for older adults in six countries (Andorra, France, Italy, Luxembourg, Spain, and the United States) were reported across 43 studies on over 1.38 million people. We found that nirsevimab uptake on population level ranged from 51.2% to 90.1% during the 2023/24 Northern Hemisphere RSV season. For RSV maternal vaccine and RSV vaccines for older adults, the meta-estimates of uptake were 30.5% and 18.2%, respectively, although all data were from the United States. Uptake data by subgroups showed differences in ethnic/racial groups and socioeconomic strata.

**Implications of all the available evidence**

Our findings highlight the need for critical reflection on eligibility criteria, maturation of implementation programmes and supply chain considerations, and targeted efforts to improve vaccine accessibility, acceptance, and equity.

## Introduction

Respiratory syncytial virus (RSV) is responsible for a common and highly contagious acute respiratory infection (ARI) around the world, with the highest RSV-associated disease burden seen in infants under 12 months of age, older adults, and adults with underlying chronic conditions and multimorbidities.^1^ RSV is transmitted via respiratory droplets or by direct contact with an infected person, and infection rates show seasonal patterns, increasing during winter months in temperate climates and during rainy seasons in tropical areas.^2^^,^^3^

A population-based serosurvey in the Netherlands showed that almost 85% of children had been infected with RSV by the time they were two years old.^4^ An estimated 4% of infants with RSV disease under 12 months of age are hospitalised, and 2–4% of these hospitalised children are admitted to intensive care units.^5^^,^^6^ Globally, it was recently (2019) estimated that 20,500 deaths in infants under 12 months old were RSV-associated, with 97% of these deaths occurring in low- and middle-income countries (LMICs).^7^

In addition to acute disease, observational studies have found an independent association between RSV-positive respiratory infection under 12 months of age and development of recurrent wheezing by the age of six years old (odds ratio (OR) 2.6 (95% CI: 1.7–4.0)) compared to children without an RSV-positive infection in the first 12 months of life.^8^ Until recently, the only pharmacological RSV preventive product widely used to reduce severe RSV-associated disease was the monoclonal antibody palivizumab. However, due to high cost, it was only indicated for infants at the highest risk of RSV-associated severe disease and required up to five monthly doses given during the RSV season.^9^^,^^10^ Children born at term and without chronic comorbidities are not eligible, despite accounting for up to 90% of RSV hospitalisations.^11^

In recent years, two novel products for the prevention of severe RSV-associated disease have become available for infants: passive immunisation with the monoclonal antibody nirsevimab and maternal vaccination during pregnancy.^12^ Nirsevimab is a humanised IgG1k class monoclonal antibody with an extended half-life, remaining effective for up to six months after a single administration. It targets the RSV fusion (F) protein, binding its pre-fusion form and preventing the virus from entering the host cells.^12^^,^^13^ In clinical trials, nirsevimab has demonstrated high efficacy of 74%–83% in reducing RSV-associated medically attended lower respiratory tract infections (LRTI) and reducing the need for supplemental oxygen by 76% in infants under 12 months old.^12^^,^^13^

The other product that has been approved for use is the maternal RSV F protein vaccine, which is recommended to pregnant individuals between 24 and 36 weeks of gestation (national vaccination recommendations vary) to induce transplacental transfer of anti-RSV antibodies.^9^ A randomised controlled trial found that the RSV maternal vaccine prevented approximately 82% of RSV-associated severe LRTI in infants up to three months old.^3^ However, the effectiveness of these products needs to be investigated in post-licensure studies to confirm the effects in real-world conditions and among individuals with diverse clinical and sociodemographic characteristics.

Moreover, RSV infection poses a substantial threat to adults 60 years of age and older who are at an increased risk of severe RSV-associated disease. Data from the United States have shown that RSV infection leads to over 172,000 hospitalisations and almost 15,000 deaths in adults aged 60 years and older, annually.^14^ In clinical trials, vaccines showed efficacy of over 80% in preventing RSV-associated LRTI in adults 60 years of age and older. However, due to strict inclusion criteria, these clinical trials did not provide efficacy estimates for some of the most vulnerable older individuals, such as adults over the age of 75 years and people with immunosuppression.^15^^,^^16^

Nirsevimab was approved for use by the European Medicines Agency on October 31, 2022, to prevent severe RSV-related disease in infants, followed by approvals in the United Kingdom, Canada, and the United States on November 9, 2022, April 19, 2023, and July 17, 2023, respectively. One RSV maternal vaccine (Abrysvo, Pfizer) has been recommended for use in pregnant individuals; three RSV vaccines (Arexvy, GSK; Abrysvo, Pfizer; mResvia, Moderna) have been authorised for use in adults aged 60 years and older. During the 2023/24 RSV season, local recommendations for the use of these products varied between countries and regions where they have been introduced. In addition to monitoring the effectiveness and safety of these interventions in real-world settings, it is essential to develop successful immunisation strategies that facilitate high uptake of nirsevimab in infants, the RSV maternal vaccine, and RSV vaccines for older adults.

Since countries in Europe and North America initiated routine RSV immunisation programmes in the general population, several studies have been published, describing country-specific immunisation strategies and subsequent uptake data. To our knowledge, uptake rates of the immunisation campaigns of the 2023/24 RSV season have not previously been systematically reviewed. We aimed to investigate the uptake of the RSV maternal vaccine, RSV vaccines for older adults, and passive immunisation with nirsevimab in infants in relation to immunisation strategies and population characteristics in countries where these products have been introduced. As more countries prepare to introduce these products, a comprehensive overview of uptake data may provide opportunities for developing successful implementation strategies and shared learning, as well as plans for addressing potential challenges with uptake, inequalities, and barriers to access.

## Methods

### Search strategy and selection criteria

For this systematic review and meta-analysis, we completed four monthly searches in Ovid Embase, Ovid MEDLINE, and Ovid Global Health databases from November 5, 2024 to February 5, 2025. To identify potentially relevant studies published between December 1, 2022 and February 5, 2025, we used search terms such as “respiratory syncytial virus”, “vaccine”, “immunisation”, and “uptake”, without language or geographical restrictions. Full search strategies are included in the Supplementary Materials (p 2). Additionally, reference lists of the included studies were hand-searched to identify any other relevant studies.

During screening, we included primary studies conducted in real-world settings, reporting uptake data for nirsevimab, the RSV maternal vaccine, or RSV vaccines for older adults. Studies without data from real-world settings, such as clinical trials, modelling studies, and cost-effectiveness studies were excluded. After removing duplicates, screening was independently conducted by two of four reviewers (DT, BL, SF, and HK) using Covidence software.^17^ Two reviewers extracted all relevant data using a pre-designed data extraction form, as well as carried out risk of bias assessments for the included studies using the relevant Joanna Briggs Institute (JBI) Critical Appraisal Tools.^18^ We extracted definitions for the target population, overall sample size, eligible population (defined as proportion of eligible individuals within the total study population), reasons for ineligibility or exclusion, and reported uptake data. Uptake data were extracted for all reported population subgroups. Information on study settings, study designs, recruitment periods, data sources, target and immunised populations, and overall socio-demographic and clinical characteristics of the study populations were recorded for all included studies. For quality assessment, studies with scores 50% and less were classified as ‘high risk’, scores between 51% and 75% were considered ‘medium risk’, and studies with scores above 75% were considered ‘low risk’. Detailed criteria and assessment scores for all studies are provided in the Supplementary Materials (p 4). Any conflicts were resolved in discussion with a third reviewer.

### Data analysis

Uptake data were calculated from the number of individuals reported eligible and number of individuals reported as immunised. For countries with available data, we compared uptake in subgroups stratified by sociodemographic or clinical characteristics, including age group, sex assigned at birth, insurance type, nationality, racial group, ethnicity, and comorbidities. Subgroup analyses of uptake were carried out to obtain pooled OR estimates. We also conducted sensitivity analyses, only selecting studies considered at ‘low risk’ of bias according to our quality assessment. To avoid duplicate data, for populations with updated uptake estimates reported in several studies over time, only the most recently published data were included in the meta-analysis.

### Statistics

Odds ratio (OR) and corresponding 95% confidence intervals were calculated by comparing uptake data across subgroups within each study. For countries with at least three population-based studies with uptake data for nirsevimab, RSV maternal vaccine or RSV vaccines for older adults, we conducted meta-analysis to provide pooled uptake estimates using random-effects modelling. Study heterogeneity was assessed with I^2^ statistic, categorised as low (<25%), moderate (25–50%), or high (>50%). Meta-analyses were performed with ‘meta’ package in software R (version 4.2.3).

### Ethics

We used published population-level data; therefore, ethical approvals were not required. A protocol for this study was registered on PROSPERO (registration number: CRD42025643585), and the study followed the Preferred Reporting Items for Systematic Reviews and Meta-Analyses (PRISMA) guidelines.^19^

### Role of the funding source

The funder was not involved in the design of this study, data collection, data analysis, interpretation, writing of the manuscript or decision to submit the study for publication.

## Results

**Fig. 1:**
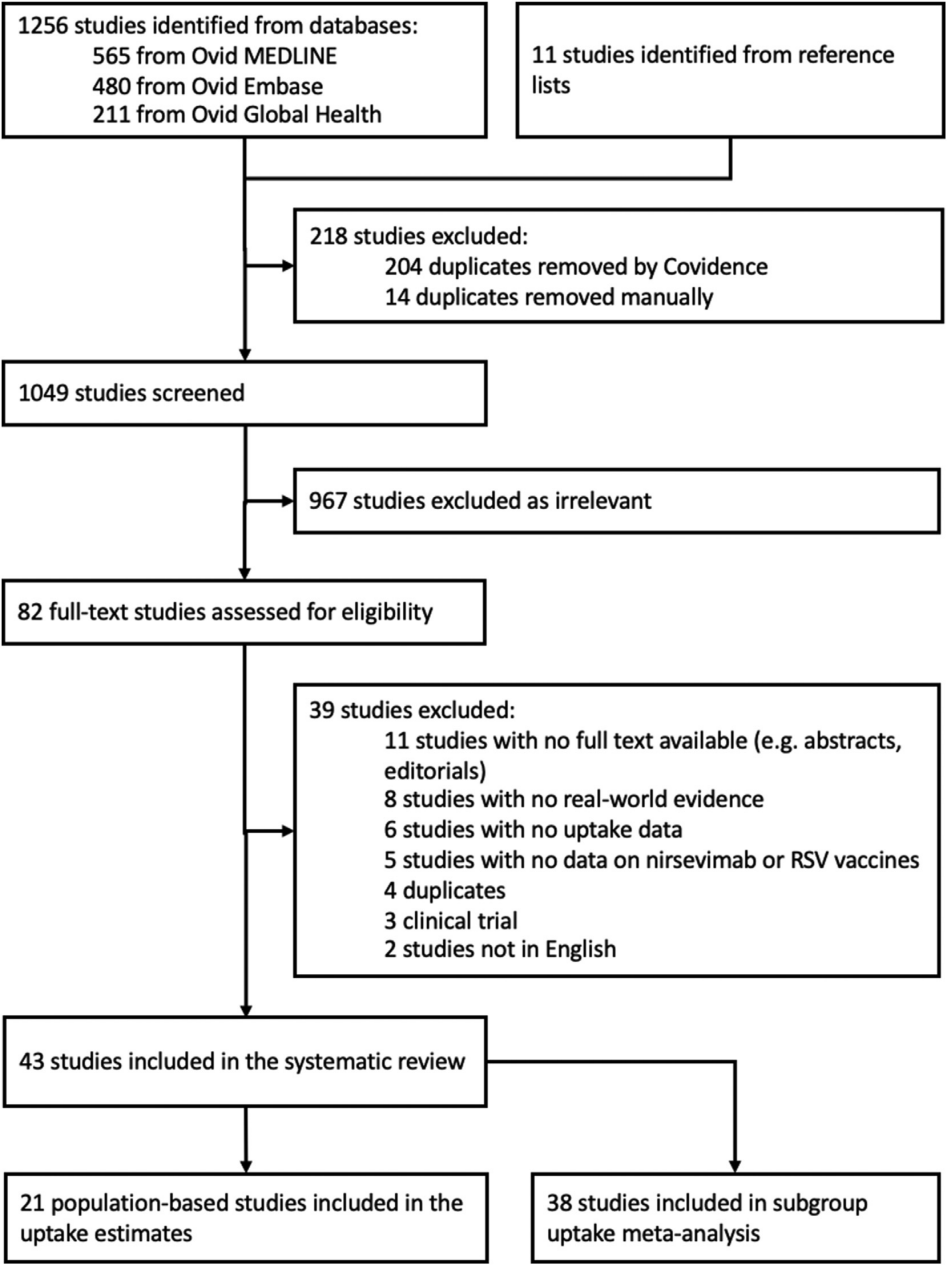
PRISMA flow chart.

A total of 1256 studies were identified from the search with 11 additional studies from reference lists as shown in the PRISMA flow chart (Fig. 1). After full-text screening, 39 studies were excluded (studies and exclusion reasons are listed in Supplementary Materials, p 3). Forty-three studies were included in the systematic review including 21 population-based studies, which contributed data to the overall uptake meta-estimates. Data from 38 of 43 studies were used in uptake subgroup meta-analysis (Supplementary Materials, p 13). The included studies represented uptake of RSV immunisation products in a sample of over 1.38 million individuals. Fourteen (33%) of the included studies were cohort studies, 14 (33%) were case-control studies, 10 (23%) were interrupted time series studies, and five (11%) studies were cross-sectional. One (2%) of the included studies was a pre-print.^20^ For 36 (87%) studies the data were sourced from electronic health records (EHRs) or medical registries, three (7%) studies used a combination of electronic health records and patient interviews, and four (9%) studies relied on data from surveys. 42 (98%) studies provided uptake data for the 2023/24 RSV season; one (2%) study focused on the 2024/25 RSV season. Detailed characteristics of the included studies are provided in Table 1. The risk-of-bias assessment scores ranged from 33% to 100%, with 19 (44%) of studies considered at ‘low risk’ of bias, 14 (33%) at ‘medium risk’, and 10 (23%) at ‘high risk’.

**Table 1:** Characteristics of the included studies.

Study	Intervention	Country	Study period	Study design	Target population for intervention	Study population	Study setting	Sample size	Population demographics
Agüera et al. (2024)	Nirsevimab	Spain, Andorra	Nov 2023–Feb 2024	Test-negative case control study	Infants <6 months old	Infants <12 months old, hospitalised with bronchiolitis	Secondary care	234	Median (IQR) age: 3.6 (1.5–8.1) months; 41% females; 73% had no comorbidities
Alejandre et al. (2024)	Nirsevimab	Spain	Oct 1, 2023–Feb 4, 2024	Interrupted time series	Infants <6 months old	Infants <10 months old, hospitalised at ICU with bronchiolitis	Secondary care	73	Median (IQR) age: 80 (40–275) days post-nirsevimab; 47% females; 68% had no comorbidities
Andina Martínez et al. (2024)	Nirsevimab	Spain	Nov 1, 2023–Jan 31, 2024	Interrupted time series	Infants <6 months old (extended catch-up); <3 months old (limited catch-up); born during RSV season (no catch-up) depending on region in Spain	Infants <6 months old, with acute bronchiolitis seen at emergency department or hospitalised	Secondary care	84,747 medical visits	NA (no population demographics provided)
Ares-Gómez et al. (2024)	Nirsevimab	Spain	Sep 25–Dec 31, 2023	Prospective, population-based longitudinal study	Children 6–24 months old with high-risk factors	6–24 months old children with high-risk conditions	Secondary care	10,259 (360 individuals were included in the meta-analysis to avoid data duplication with Mallah et al. (2024))	Median (IQR) age: 4.0 (2.0–6.0) months; 49% females
Assad et al. (2024)	Nirsevimab	France	Oct 15–Dec 10, 2023	Prospective matched case-control study	Infants <8 months old (newborns prioritised due to shortages)	Infants <12 months old, hospitalised with RSV-associated ARI	Secondary care	1035	Median (IQR) age for cases: 3.1 (1.8–5.3) months, median (IQR) age for controls: 3.4 (1.6–5.6) months; 46% females
Barbas Del Buey et al. (2024)	Nirsevimab	Spain	Oct 1, 2023–Feb 29, 2024	Prospective cohort study	Infants <6 months old	Infants <10 months old	Primary & secondary care	37,067	Median (IQR) age: 6.26 (4.46) months; 48% females
Birabaharan et al. (2024)	RSV vaccine for older adults	USA	May 3, 2023–Oct 9, 2024	Cohort study	Adults ≥60 years old	Adults ≥60 years old	Community	357,814	Mean (SD) age: 72.5 (7.1) years for cases, 69.7 (8.1) years for controls; 58% females
Cantais et al. (2024)	Nirsevimab	France	Sep 2023–Feb 2024	Interrupted time series	Newborns at maternity wards born after Sep 15, 2023 (because of shortages)	Infants <6 months old	Primary & secondary care	748	NA (no population demographics provided)
Carbajal et al. (2024)	Nirsevimab	France	Oct 14, 2023–Feb 29, 2024	Case control study	Infants <8 months old (newborns prioritised due to shortages)	Infants <12 months old seen at emergency departments	Secondary care	2786	43.7% females
Chauvel et al. (2024)	Nirsevimab	France	Sep 15–Dec 31, 2023	Interrupted time series	Newborns at maternity wards (because of shortages)	Infants <6 months old	Secondary care	83	Median (IQR) age: 76 (40–110) days; 54% females
Coma et al. (2024)	Nirsevimab	Spain	Oct 1, 2023–Jan 31, 2024	Retrospective cohort study	Infants <6 months old	<9 months old, hospitalised with RSV-associated LRTI	Primary & secondary care	26,525	Median (IQR) age: 88 (44–134) days for immunised, 106 (52–151) days for non-immunised; 49% females
Consolati et al. (2024)	Nirsevimab	Italy	May 1, 2023–Feb 15, 2024	Interrupted time series	Infants <8 months old	Infants born between May 1, 2023 and Feb 15, 2024	Primary & secondary care	537	NA (no population demographics provided)
Ernst et al. (2024)	Nirsevimab	Luxembourg	Sep 24–Dec 31, 2023	Interrupted time series	Infants <9 months old; children <2 years old at high risk for severe respiratory infection	Children <3 years old, hospitalised with RSV-associated ARI	Secondary care	630	Mean (SD) age: 14.4 (12.9) months
Espeleta-Fox et al. (2024)	Nirsevimab	Spain	Nov 2023–Mar 2024	Interrupted time series	Infants <6 months old	Infants <12 months old, hospitalised at ICU with RSV-associated LRTI	Secondary care	435	Median (IQR) age: 235.5 (183) days for controls, 113 (186) days for cases; 43% females
Estrella-Porter et al. (2024)	Nirsevimab	Spain	Oct 1, 2023–Jan 9, 2024	Retrospective observational study	Infants <6 months old	All infants <6 months old (born between Apr 1, 2023 and Jan 7, 2024)	Primary & secondary care	27,362	49% females
Ezpeleta et al. (2024)	Nirsevimab	Spain	Oct 1, 2023–Jan 28, 2024	Population-based cohort study	Newborns at maternity wards	Infants <3 months old in community; infants <4 months old, hospitalised, at emergency department, or admitted to ICU due to RSV-associated LRTI	Secondary care	1177	Median (IQR) age: 38.5 (14–60) days; 46% females
Geng et al. (2024)	RSV vaccine for older adults	USA	Jan 9–Mar 4, 2024	Cross-sectional study	Adults ≥60 years old	Adults ≥60 years old	Primary care	49,322	53% females
Homo et al. (2024)	Nirsevimab	USA	Oct 16, 2023–Feb 29, 2024	Cohort study	Newborns at a maternity ward of a military treatment centre	Newborns at a maternity ward of a military treatment centre (born between Oct 16, 2023 and Feb 29, 2024)	Community	500 (289 were eligible for nirsevimab as the rest were immunised via RSV maternal vaccine)	Median (IQR) maternal age: 30 (8) years; 48% of infants female
Homo et al. (2024)	RSV maternal vaccine	USA	Oct 16, 2023–Feb 29, 2024	Cohort study	Pregnant individuals between 32 weeks 0 days and 36 weeks 6 days gestation	Pregnant individuals between 32 weeks 0 days and 36 weeks 6 days gestation	Tertiary care	(as above)	(as above)
Jimeno Ruiz et al. (2024)	Nirsevimab	Spain	Oct 1, 2023–Mar 31, 2024	Retrospective observational study	Infants <6 months old	Infants <12 months old, hospitalised with RSV-associated ARI	Secondary care	77	Mean (SD) age: 6.4 ( ± 4.3) months; 53% females
Kemp et al. (2025)	Nirsevimab	USA	Oct 1, 2023–Mar 31, 2024	Cross-sectional study	Newborns at maternity wards	Infants born between Oct 1, 2023 and Mar 31, 2024	Community	27,788 (birthing parents)	48% of infants female
Kemp et al. (2025)	RSV maternal vaccine	USA	Sep 1, 2023–Jan 30, 2024	Cross-sectional study	Pregnant individuals between 32 weeks 0 days and 36 weeks 6 days gestation	Pregnant individuals between 32 weeks 0 days and 36 weeks 6 days gestation	Community	(as above)	Maternal age groups (years): <20 (2.3%), 20–24 (13.9%), 25–29 (27.7%), 30–34 (34.0%), 35–39 (17.9%), 40–44 (4.0%), ≥45 (0.2%)
Lefferts et al. (2024)	Nirsevimab	USA	Oct 23, 2023–Jun 30, 2024	Test-negative case control study	American Indian or Alaska Native children <20 months old	American Indian or Alaska Native children <28 months old with medically-attended ARI	Secondary care	472	Median (IQR) age: 9 (0–27) months; 47% females
Lenglart et al. (2025)	Nirsevimab	France	Oct 1, 2023–Feb 29, 2024	Test-negative case control study	Infants <8 months old (newborns prioritised due to shortages)	Infants <12 months old, seen at emergency department with first bronchiolitis	Secondary care	383	Median (IQR) age: 3.3 (2.0–5.5) months for cases, 2.0 (1.0–4.0) months for controls; 43% females
Levy et al. (2024)	Nirsevimab	France	Sep 15, 2023–Jan 15, 2024	Interrupted time series	Infants <8 months old (newborns prioritised due to shortages)	Infants <12 months old, at primary care with RSV-related bronchiolitis	Primary care	399	NA (no population demographics provided)
López-Lacort et al. (2024)	Nirsevimab	Spain	Oct 1, 2023–Jan 10, 2024	Screening design and test negative case-control study	Infants <6 months old	Infants <9 months old, hospitalised with LRTI	Secondary care	15,676 (166 individuals were included in the meta-analysis to avoid data duplication with Estrella-Porter et al. (2024))	44% under 3 months old
López-Lacort et al. (2025)	Nirsevimab	Spain	Nov 1, 2023–Feb 29, 2024	Test-negative case control study	Infants <6 months old	Infants <10 months old, at primary care with LRTI	Primary care	160	Median (IQR) age: 4.5 (3.0–6.0) months; 36% females; 11% born preterm (<37 weeks gestation)
Mallah et al. (2024)	Nirsevimab	Spain	Sep 25, 2023–Apr 15, 2024	Prospective, population-based longitudinal study	Infants <6 months old	Infants <12 months old (born between Mar 31, 2023 and Mar 31, 2024)	Community	14,476	Median (IQR) age: 6.7 (3.7–9.6) months; 49% females; 7% born preterm (<37 weeks of gestation)
Martinon-Torres et al. (2023)	Nirsevimab	Spain	Sep 25–Oct 15, 2023	Prospective, population-based longitudinal study	Infants <6 months old	Infants <12 months old (born between Mar 31, 2023 and Apr 1, 2024)	Community	8667 (these individuals were not included in the meta-analysis to avoid data duplication with Mallah et al. (2024))	NA (no population demographics provided)
Molina Gutiérrez et al. (2024)	Nirsevimab	Spain	Oct 2–Dec 31, 2023	Interrupted time series	Infants <6 months old	Infants <9 months old, seen at emergency department with RSV-related ARI	Secondary care	52	37% females
Moline et al. (2024a)	Nirsevimab	USA	Oct 1, 2023–Feb 27, 2024	Test-negative case control study	Infants <8 months old	Children <13 months, hospitalised with ARI	Secondary care	699	42% females; 21% preterm (born <37 weeks of gestation); 6% with a high-risk condition
Moline et al. (2024b)	Nirsevimab	USA	Sep 1, 2023–Apr 30, 2024	Test-negative case control study	Infants <8 months old	Infants <12 months old with medically attended ARI	Primary & secondary care	28,689 (2989 for 2023–24 season)	Median (IQR) age: 15 (6–29) months; 43% females
Moline et al. (2024b)	RSV maternal vaccine	USA	Sep 1, 2023–Apr 30, 2024	Test-negative case control study	Pregnant individuals between 32 weeks 0 days and 36 weeks 6 days gestation	Infants <12 months old with medically-attended ARI	Primary & secondary care	(as above)	(as above)
Motta et al. (2025)	RSV vaccine for older adults	USA	Oct 20–Nov 6, 2023	Cross-sectional study	Adults ≥60 years old	Adults ≥60 years old	Community	358	Mean (SD) age: 69.4 (6.7) years; 55% females
Paireau et al. (2024)	Nirsevimab	France	Sep 15, 2023–Jan 31, 2024	Test-negative case control study	Infants <8 months old (newborns prioritised due to shortages)	Infants <4.5 months old, admitted to ICU with bronchiolitis; infants <9.5 months with comorbidities	Secondary care	288	91% aged 0–3 months, 9% aged 4–8 months; 45% females; 88% ad no comorbidities
Payne et al. (2024)	RSV vaccine for older adults	USA	Oct 1, 2023–Mar 31, 2024	Test-negative case control study	Adults ≥60 years old	Adults ≥60 years old hospitalised or at emergency department with LRTI	Secondary care	36,706 hospitalisations	Median (IQR) age: 76 (69–84) years at hospitalisation, 75 (67–82) years at emergency department visit; 53% females hospitalised, 55% females at emergency department
Pérez Martín and Zornoza Moreno (2024)	Nirsevimab	Spain	Sep 25, 2023–Apr 8, 2024	Cross-sectional study	Infants <6 months old; children <24 months old at high-risk of severe respiratory infection; infants <12 months old if born premature (<34 weeks' gestation)	<6 months old (born between Apr 1, 2023 and Mar 31, 2024)	Community	13,086	49% females
Perramon-Malavez et al. (2025)	Nirsevimab	Spain	Oct 1, 2023–Jan 31, 2024	Interrupted time series	Newborns at maternity wards	Infants <4 months old	Community	15,341	Median (IQR) age: 69 (10–122) days for immunised, 60.5 (10–122) for non-immunised; 48% females
Puckett et al. (2025)	Nirsevimab	USA	Oct 24, 2023–Apr 1, 2024	Retrospective observational study	Infants <8 months old; children <19 months old with comorbidities	Infants <8 months old; children <19 months old with comorbidities	Community	1110	99.9% infants aged under 8 months old; 52% females
Razzaghi et al. (2024)	Nirsevimab	USA	Sep 1, 2023–Jan 31, 2024	Cross-sectional study	Infants <8 months old	Infants <8 months old (eligible to receive nirsevimab between Oct 1, 2023 and Mar 31, 2024)	Community	866	NA (no population demographics provided)
Razzaghi et al. (2024)	RSV maternal vaccine	USA	Oct 1, 2023–Mar 31, 2024	Cross-sectional study	Pregnant individuals between 32 weeks 0 days and 36 weeks 6 days gestation	Pregnant individuals between 32 weeks 0 days and 36 weeks 6 days gestation any time during Sep 1, 2023–Jan 31, 2024	Community	678 (pregnant individuals)	NA (no population demographics provided)
Reses et al. (2023)	RSV vaccine for older adults	USA	2023/24 season (till Dec 10, 2023)	Surveillance study	Adults ≥60 years old	Nursing home residents	Nursing homes	238,449 (not included in the total sample size to avoid data duplication with Reses et al. (2024))	NA (no population demographics provided)
Reses et al. (2024)	RSV vaccine for older adults	USA	2024/25 season (till Nov 10, 2024)	Surveillance study	Adults ≥60 years old	Nursing home residents	Nursing homes	661,075	NA (no population demographics provided)
Son et al. (2024)	RSV maternal vaccine	USA	Sep 22, 2023–Jan 31, 2024	Retrospective cohort study	Pregnant individuals between 32 weeks 0 days and 36 weeks 6 days gestation	Pregnant individuals who gave birth to singleton gestations at 32 weeks' gestation or later from Sep 22, 2023 to Jan 31, 2024	Community	2973	Maternal median (IQR) age: 34.9 (32.4–37.7) years
Surie et al. (2024)	RSV vaccine for older adults	USA	Oct 1, 2023–Mar 31, 2024	Test negative case control study	Adults ≥60 years old	Adults ≥60 years old, hospitalised due to ARI	Secondary care	2978	Median (IQR) age: 72 (66–80) years; 51% females; 24% immunocompromised; 96% chronic condition
Tartof et al. (2024)	RSV vaccine for older adults	USA	Nov 24, 2023–Apr 9, 2024	Test-negative case control study	Adults ≥60 years old	Adults ≥60 years old hospitalised or at emergency department with LRTI	Secondary care	7047	Mean (SD): 76.8 (9.6) years; 43% aged 60–74 years, 57% aged 75 or more years; 54% females
Xu et al. (2024)	Nirsevimab	USA	Oct 1, 2023–May 9, 2024	Test negative case-control study	Infants <8 months old; infants 8–12 months old with a risk factor for severe respiratory infection	Children <19 months old with medically attended RSV-associated ARI	Primary & secondary care	3090	Median (IQR) age: 6.7 (3.6–9.7) months; 43% females

RSV = respiratory syncytial virus; ARI = acute respiratory infection; LRTI = lower respiratory tract infection; ICU = intensive care unit; IQR = interquartile range; SD = standard deviation. NA = not available.

Uptake of nirsevimab was reported in 34 studies from six different countries, including 16 studies from Spain,^21^, ^22^, ^23^, ^24^, ^25^, ^26^, ^27^, ^28^, ^29^, ^30^, ^31^, ^32^, ^33^, ^34^, ^35^, ^36^ eight studies from the United States,^20^^,^^37^, ^38^, ^39^, ^40^, ^41^, ^42^, ^43^ seven studies from France,^44^, ^45^, ^46^, ^47^, ^48^, ^49^, ^50^ one study from Italy,^51^ one study with combined data from Andorra and Catalonia (Spain),^52^ and one study from Luxembourg.^53^ Of these, eight studies from Spain, five studies from the United States, and one study from each of France, Luxembourg, and Italy were population-based. Five studies reported uptake of the RSV maternal vaccine in the United States^38^, ^39^, ^40^^,^^42^^,^^54^; four of which were population-based studies. Eight studies, including four population-based studies, reported data on the RSV vaccines for older adults in the United States.^16^^,^^55^, ^56^, ^57^, ^58^, ^59^, ^60^, ^61^

### Nirsevimab

**Fig. 2:**
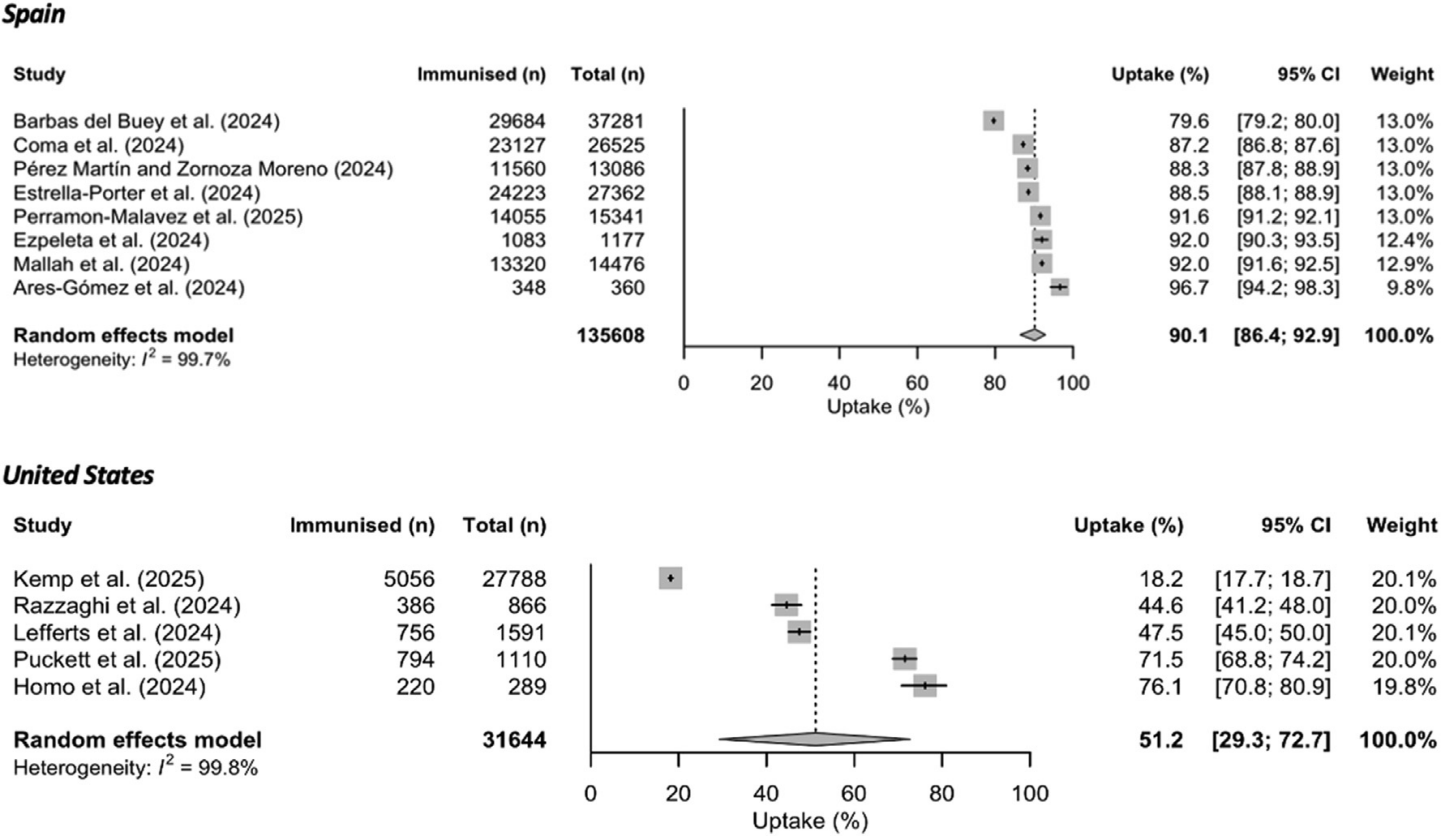
Uptake (%) of nirsevimab among eligible children in Spain and the United States during the 2023/24 RSV season. 95% CI = 95% confidence interval (shown with error bars); n = number of individuals.

The overall estimated uptake of nirsevimab in Spain on population level during the 2023/24 RSV season was 90.1% (95% CI: 86.4–92.9) (Fig. 2). Subgroup meta-analyses were stratified by immunisation roll-out group (seasonal, catch-up), sex assigned at birth, nationality (Spanish, non-Spanish), and gestational age (37 or more weeks, under 37 weeks) (Table 2). Higher uptake was seen in the seasonal group (children born after the start of the immunisation campaign; OR 2.13 (95% CI: 1.50–3.03)) compared to children immunised as part of the catch-up group (born between April 1, 2023, and the start of the immunisation campaign). Uptake of nirsevimab was significantly higher in children with Spanish nationality (OR 1.69; 95% CI: 1.41–2.03) compared to non-Spanish children.

**Table 2:** Meta-analysis of RSV immunisation product uptake stratified by subgroups.

Product	Variable	Subgroup	Reference	No. of studies	Odds ratio (95% CI)	I^2^
Nirsevimab (Spain)	Immunisation enrolment group	Seasonal	Catch-up	3	2.13 (1.50–3.03)	91.8%
	Sex	Males	Females	10	1.00 (0.95–1.04)	25.8%
	Gestational age	Preterm (<37 weeks)	Term (≥37 weeks)	3	1.12 (0.94–1.33)	86.9%
	Nationality	Spanish	Non-Spanish	4	1.69 (1.41–2.03)	93.4%
Nirsevimab (United States)	Sex	Males	Females	6	0.95 (0.90–1.01)	0.0%
	Gestational age	Preterm (<37 weeks)	Term (≥37 weeks)	5	1.74 (1.29–2.35)	61.4%
	Comorbidities	Yes	No	5	3.36 (1.31–8.64)	93.1%
	Health insurance type	Public	Private or military	6	1.18 (1.01–1.37)	51.0%
		Private or military	None/other	6	2.35 (0.98–5.66)	70.7%
		Public	None/other	6	2.66 (1.24–5.71)	65.8%
	Residential area	Rural	Urban	3	1.26 (0.43–3.70)	97.7%
	Race and ethnicity	Black or African American (non-Hispanic)	White (non-Hispanic)	5	1.32 (0.97–1.81)	75.2%
		Asian (non-Hispanic)	White (non-Hispanic)	4	1.11 (0.80–1.53)	47.2%
		Hispanic	White (non-Hispanic)	6	1.43 (1.22–1.67)	38.7%
		American Indian or Alaska Native (non-Hispanic)	White (non-Hispanic)	3	1.25 (0.91–1.72)	0.0%
		Multiple/other (non-Hispanic)	White (non-Hispanic)	6	1.09 (0.77–1.53)	64.3%
		Hispanic	Non-Hispanic	6	1.26 (1.02–1.55)	72.0%
Nirsevimab (France)	Age group	<3 months	3–12 months	5	4.60 (3.58–5.92)	43.4%
RSV maternal vaccine (United States)	Health insurance type	Public	Private or military	4	0.38 (0.24–0.61)	90.9%
		None/other	Private or military	3	0.26 (0.08–0.82)	75.5%
		None/other	Public	3	0.51 (0.07–3.70)	89.2%
	Race and ethnicity	Black or African American (non-Hispanic)	White (non-Hispanic)	4	0.61 (0.41–0.90)	71.4%
		Asian (non-Hispanic)	White (non-Hispanic)	3	1.45 (0.75–2.81)	92.7%
		Hispanic	White (non-Hispanic)	4	0.62 (0.40–0.96)	82.6%
		Multiple/other (non-Hispanic)	White (non-Hispanic)	4	0.78 (0.69–0.89)	0.0%
		Hispanic	Non-Hispanic	4	0.66 (0.44–0.99)	81.2%
RSV vaccines for older adults (United States)	Age group	≥75 years	60–74 years	4	1.69 (1.49–1.92)	85.3%
	Sex	Males	Females	5	1.00 (0.95–1.06)	71.7%
	Immuno-compromised status	Yes	No	3	1.22 (1.02–1.47)	49.9%
	Comorbidities	Yes	No	3	1.56 (1.31–1.85)	0.0%
	Race and ethnicity	Black or African American (non-Hispanic)	White (non-Hispanic)	5	0.50 (0.33–0.75)	96.1%
		Asian (non-Hispanic)	White (non-Hispanic)	3	0.85 (0.45–1.62)	98.5%
		Hispanic	White (non-Hispanic)	5	0.54 (0.43–0.67)	91.8%
		Multiple/other (non-Hispanic)	White (non-Hispanic)	5	0.75 (0.62–0.92)	78.9%
		Hispanic	Non-Hispanic	5	0.58 (0.48–0.69)	88.5%

95% CI = 95% confidence interval.

In the United States, the overall uptake of nirsevimab during 2023/24 RSV season pooled from five population-based studies was 51.2% (95% CI: 29.3–72.7) (Fig. 2). Subgroup analyses showed that uptake was higher in those with at least one comorbidity (OR 3.36; 95% CI: 1.31–8.64), preterm infants (OR 1.74; 95% CI: 1.29–2.35), and Hispanic populations (ORs 1.43 (95% CI: 1.22–1.67) and 1.26 (95% CI: 1.02–1.55) compared to White (non-Hispanic) populations and non-Hispanic populations, respectively). Uptake was found to be higher in children with public insurance compared to no or other insurance (OR 2.66; 95% CI: 1.24–5.71).

Uptake data on nirsevimab in France during the 2023/24 RSV season were from seven studies: six were conducted in children attending primary or secondary care with ARI and one population level study with reported uptake of 76.5%.^44^ Subgroup analysis showed that the uptake was higher in infants under three months old (OR 4.60; 95% CI: 3.58–5.92) compared to infants 3–12 months old. One study from Luxembourg reported population level uptake of 83.8%,^53^ and one study from Valle d'Aosta (Italy) reported overall uptake of 68.7%.^51^ One study with combined data from Andorra and Catalonia (Spain) reported uptake of 60.2% (in patients hospitalised with bronchiolitis).^52^

Moreover, our pooled subgroup estimates showed that lower uptake was associated with having RSV-related or all-cause ARI (Supplementary Materials, p 10). In Spain, children with medically attended RSV-associated ARI had a significantly lower uptake of nirsevimab (27.5%; 95% CI: 18.4–39.1) compared to children with all-cause ARI (71.4%; 95% CI: 53.9–84.1) and all children within the population (90.1%; 95% CI: 86.4–92.9). Similarly, the pooled estimates in the United States showed that children with medically attended RSV-associated ARI had a significantly lower uptake of nirsevimab (2.9%; 95% CI: 0.7–11.2) compared to all children within the population (51.2%; 95% CI: 29.3–72.7). For France, most uptake data came from children presenting with RSV-associated ARI and all-cause ARI with pooled uptake estimates of 20.5% (95% CI: 6.1–50.7) and 20.4% (95% CI: 18.5–22.5), respectively, which were lower than the population level estimate of 76.5% (one study).^44^

### Maternal vaccine

**Fig. 3:**
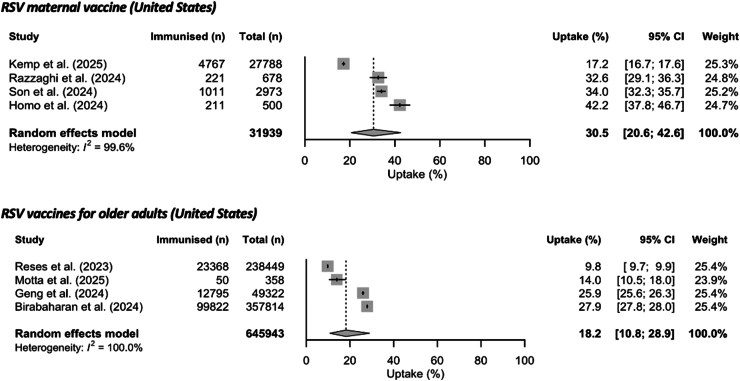
Uptake (%) of RSV maternal vaccine during pregnancy (between 32 and 36 weeks of gestation) and RSV vaccines in eligible older adults in the United States during the 2023/24 RSV season. 95% CI = 95% confidence interval (shown with error bars); n = number of individuals.

Pooled uptake of RSV maternal vaccines among pregnant individuals in the United States was 30.5% (95% CI: 20.6–42.6) (Fig. 3). Subgroup meta-analyses found a significantly lower uptake of the RSV maternal vaccine in individuals without health insurance (OR 0.26; 95% CI: 0.08–0.82) or with public insurance (OR 0.38 95% CI: 0.24–0.61) compared to people with private or military insurance. Uptake estimates were significantly lower in Black or African American (non-Hispanic) (OR 0.61; 95% CI: 0.41–0.90) and Hispanic (OR 0.62; 95% CI: 0.40–0.96) ethnic groups compared to people in White (non-Hispanic) groups.

### Vaccines for older adults

Overall uptake of RSV vaccines among adults aged 60 years and older pooled from four population-based studies reporting data for the 2023/24 RSV season was 18.2% (95% CI: 10.8–28.9) as shown in Fig. 3. Our subgroup analyses showed that uptake was higher in those aged 75 years or more (OR 1.69; 95% CI: 1.49–1.92), with at least one comorbidity (OR 1.56; 95% CI: 1.31–1.85), or with immunocompromised status (OR 1.22; 95% CI: 1.02–1.47). However, uptake was estimated to be lower in Black or African American (non-Hispanic) (OR 0.50; 95% CI: 0.33–0.75) and Hispanic (OR 0.54; 95% CI: 0.43–0.67) ethnic groups compared to White (non-Hispanic) groups.

## Discussion

Our systematic review and meta-analysis found marked variations in overall uptake estimates for nirsevimab during the 2023/24 RSV season in countries with available population level data, ranging from 51.2% in the United States to 90.1% in Spain. In the United States, estimated uptake of the RSV maternal vaccine among eligible pregnant individuals and uptake of the RSV vaccines for older adults were low: 30.5% and 18.2%, respectively.

Our estimates varied widely across study sites. The reasons for between- and within-country differences in uptake are multifaceted and could reflect different healthcare systems and vaccination service delivery models, demographic make-up, attitudes or beliefs, health seeking behaviours, promotion, visibility of immunisation programmes, and target populations of the immunisation campaigns. For nirsevimab, the target populations of the immunisation campaigns varied between countries, potentially leading to differences in uptake. In Spain, nirsevimab was recommended for use in infants under six months of age on October 1, 2023, infants born during the RSV season (between October 1, 2023 and March 31, 2024), and children under two years old at increased risk of severe RSV-associated disease. However, local recommendations varied among autonomous communities in Spain. For instance, in Navarre (Spain), nirsevimab was only offered to infants born between October 1, 2023 and March 31, 2024; in Madrid (Spain), it was only available by appointment at five of 25 public hospitals instead of primary care centres and maternity wards as in other regions.^21^ In France, the immunisation campaign started on September 15, 2023, and nirsevimab was recommended for use in infants under eight months old (born after February 6, 2023). However, the recommendations were changed due to supply issues and by September 26, 2023, it was only offered to newborns in maternity wards.^49^ Our subgroup meta-analysis estimated a higher uptake in infants under three months old compared to 3–12 months old children, reflecting this policy in France (Table 2). Similarly, in the United States, recommendations were changed from infants under eight months old to under six months old due to product shortages early in the season.^37^ Therefore, the probability of receiving nirsevimab was dependent on the geographic location, infants' date of birth, and age. In Spain, significantly higher uptake was estimated in children born after the start of the immunisation campaign (seasonal group) compared to children who were under six months old on October 1, 2023 (catch-up group). This difference may be attributed to lower accessibility for infants whose caretakers had to make a separate appointment at a healthcare centre compared to being offered nirsevimab after birth at maternity wards. A delay in immunisation of an estimated 27 days was seen in infants born after October 1, 2023, whose caretakers opted not to immunise at maternity wards.^35^ These findings highlight the need to develop programmes that allow for ease of access and offer immunisation as soon after birth or at the start of the RSV seasons as possible. Additionally, consistent study methodologies looking at uptake are needed to facilitate comparisons between regions and countries and to help identify groups that need to be targeted to improve uptake.

In Spain and the United States, higher immunisation rates were seen in infants who did not have RSV-associated ARI at the time the data were collected. This potentially highlights the protective effect of nirsevimab in preventing severe disease; however, it is noted that RSV testing was usually only done at hospital in infants with severe symptoms.^49^ Therefore, the findings do not indicate whether nirsevimab also impacted the incidence of mild and moderate RSV-related disease. However, data from Spain, from a study included in this review, suggest that nirsevimab was associated with an absolute reduction in Emergency Department attendances for infants with bronchiolitis.^21^ Uptake data in studies conducted in France were mostly sourced from infants with ARI; therefore, we could not provide a meta-estimate of nirsevimab uptake in the general population.

Subgroup meta-analyses found significant differences in uptake of RSV preventive products by racial and ethnic groups in the United States. In Spain, the uptake of nirsevimab was significantly higher in Spanish children compared to non-Spanish and immigrant children. These findings highlight the need to develop effective channels for communication with groups whose first language may be different from the official language and building trust in these communities, as well as investigating other potential reasons such as barriers to access (e.g., needing to have ID and to pay), and differences in attitudes and beliefs regarding immunisation.

The uptake estimates of the RSV maternal vaccine and RSV vaccines for older adults in the United States were low. Several studies have attributed the low uptake to the lack of awareness among vaccine recipients and their healthcare providers, high costs, misinformation, vaccine hesitancy, and issues with healthcare access.^39^^,^^54^^,^^56^^,^^57^ Uptake of the RSV maternal vaccines in the United States were significantly lower in people without health insurance compared to those with private or military health insurance. Further investigation in disparities by variables related to socio-economic status is warranted.

Key strengths of our comprehensive systematic review include that we investigated uptake of three novel RSV immunisation products intended for the prevention of RSV-associated severe disease in the most vulnerable individuals, using data from 43 studies with a large total sample size of over 1.38 million individuals. We were able to carry out key subgroup meta-analyses, identifying subgroups with lower uptake. The studies included were published between December 22, 2023 and February 1, 2025, offering recent and up-to-date evidence to help inform immunisation strategies for the next RSV seasons.

However, our study has several limitations. First, the field is dynamic with several studies published every month; therefore, regularly updated evidence will be needed as more countries introduce these interventions and studies are published. Continued monitoring of emerging evidence as part of this systematic review will allow us to promptly synthesise data on RSV immunisation product uptake for the 2024/25 RSV season. Additionally, we have created a website to keep a record of updates to the review (available here: https://v1xerunt.github.io/VaccineUptake/, data are available in the Supplementary Materials, p 34). Second, our review included studies published in peer-reviewed journals and did not include uptake data published in official reports from health care institutions and public health agencies. For instance, In the United States, The Centers for Disease Control and Prevention (CDC) overall coverage estimate for nirsevimab in infants under eight months old was 41.3% in March 2024, RSV maternal vaccine coverage was 17.8% (as of January 31, 2024), and RSV vaccine coverage estimate in adults aged 65 years or older who were among Medicare Plan D recipients was 21.0% (as of June 29, 2024).^62^, ^63^, ^64^ During the 2024/25 RSV season, UK Health Security Agency (UKHSA) has reported that as of January 31, 2025, the overall vaccine coverage in the catch-up cohort (adults aged 75–79 before the programme start date) reached 50.8% in England while maternal vaccine uptake was estimated at 39.4% in October 2024.^65^ The uptake of RSV vaccine for older adults was estimated 68.6% in Scotland as of November 2024.^66^ Third, limited data were available for meta-analyses and sensitivity analyses. The 95% CIs were wide, particularly for studies with smaller sample sizes.^39^^,^^40^^,^^58^ Additionally, our pooled population-based uptake estimates showed high heterogeneity, as assessed using the I^2^ statistic (Fig. 2, Fig. 3). This may have had several reasons including differences in immunisation strategies within countries due to varying regional or local recommendations or supply issues, differences in study designs, target populations and data collection periods in relation to the RSV season (e.g., mid-season and end-season estimates). Fourth, quality of the included studies varied with some reporting insufficient detail on the study population characteristics, a lack of strategies for dealing with confounding factors, and unclear length and sufficiency of the follow-up time. However, our sensitivity analysis only focussing on studies with low risk of bias showed similar results (Supplementary Materials, p 9). Fifth, our subgroup analyses were only possible from univariable analysis and included a limited number of studies; therefore, p-values from subgroup analyses in Supplementary Materials (p 13–33) should be interpreted with caution. As more studies are published in the future, more detailed subgroup analyses accounting for potential confounders will be possible. Sixth, the studies with uptake data on nirsevimab, RSV maternal vaccines and RSV vaccines for older adults have so far been conducted only in a few high-income countries and only over one (initial) season when the likelihood of uptake will vary considerably over that short timeframe as is common for new immunisation programmes. This limits the generalisability of our findings in other contexts and populations, including LMICs, which have the highest burden of RSV-associated severe disease.^7^

Despite these limitations, this study provides a comprehensive overview of uptake of nirsevimab, RSV maternal vaccine, and RSV vaccines for older adults. Uptake of nirsevimab varies across countries. The low uptake for both RSV maternal vaccine and RSV vaccines for older adults require further actions to be taken to protect as many people as possible.

## Contributors

TS conceptualised the study with input from DT, TW, and AS. DT, BL, SF, and HK contributed to data collection. DT led data analysis with input from BL, SF, HK, and BG. DT wrote the codes for analysis with support from BG. TS, TW, AS, JS, JB, SD, and CG led the data interpretation. JG, LM, and HX led the website building with all the information and data from this study. DT wrote the first draft of the manuscript with input from TS and BL. All authors contributed to data interpretation and critically revised the manuscript. All authors read and approved the final version of the manuscript. DT and TS had full access to and verified the underlying data of the study. All authors had final responsibility for the decision to submit for publication.

## Data sharing statement

Aggregated study data on RSV immunisation product uptake are freely accessible at https://v1xerunt.github.io/VaccineUptake/.

## Declaration of interests

JS has participated in Sanofi advisory board on nirsevimab in 2022, and participated in Allergy Therapeutics advisory board on immunotherapy. JS has received research grant from UKRI Medical Research Council. JS has received support from ALK for attending meetings and/or travel. JB has received research funding from AstraZeneca, paid to his institution. AS acknowledges the support of Health Data Research UK. DT is funded by Health Data Research UK, Inflammation and Immunity Driver Programme. BL has received Asthma + Lung UK Early Career Researcher grant (WHRD_PB1062). BG has received the studentship award from the Health Data Research UK-The Alan Turing Institute Wellcome PhD Programme in Health Data Science (Grant Ref: 218529/Z/19/Z). SD has received investigator led grant from MSD in relation to RSV, paid to his institution. SD has received support from Sanofi for attending meetings and/or travel. SD has provided consultancy and/or investigator roles in relation to product development for Janssen, AstraZeneca, Pfizer, Moderna, Valneva, MSD, iLiAD, MundiPharma, and Sanofi, paid to his institution. SD is a member of the UK Department of Health and Social Care's (DHSC) Joint Committee on Vaccination and Immunisation (JCVI) RSV subcommittee and Medicines and Healthcare products Regulatory Agency's (MHRA) Paediatric Medicine Expert Advisory Group (PMEAG). TW has received research funding from Respiratory Syncytial Virus Consortium in Europe (RESCEU), Wellcome Trust, Imperial College London, and National Institute for Health Research. All other authors declare no competing interests.
