# Supplementary material for: Real-world uptake of nirsevimab, RSV maternal vaccine, and RSV vaccines for older adults: a systematic review and meta-analysis
Source: eClinicalMedicine. 2025 Jun 3;84:103281. doi: 10.1016/j.eclinm.2025.103281 (PMC12167775; doi:10.1016/j.eclinm.2025.103281)
Supplement: Supplementary Materials [file mmc1.docx]

**Supplementary materials for “Real-world uptake of nirsevimab, RSV maternal vaccine, and RSV vaccines for older adults: a systematic review and meta-analysis”**

**Contents**

[Supplement 1: Search strategies 2](#_Toc192278538)

[Supplement 2: Excluded studies 3](#_Toc192278539)

[Supplement 3: ‘Risk of bias’ quality assessment 4](#_Toc192278540)

[Supplement 4: Sensitivity analysis 9](#_Toc192278541)

[Supplement 5: Uptake of RSV immunisation products stratified by health status at data collection 10](#_Toc192278542)

[Supplement 6: Subgroup meta-analysis for data reported in Table 2 13](#_Toc192278543)

[1. Uptake of nirsevimab in Spain 13](#_Toc192278544)

[2. Uptake of nirsevimab in France 16](#_Toc192278545)

[3. Uptake of nirsevimab in the United States 17](#_Toc192278546)

[4. Uptake of RSV maternal vaccine in the United States 24](#_Toc192278547)

[5. Uptake of RSV vaccines for older adults in the United States 29](#_Toc192278548)

[Supplement 7: Data used to create 'Uptake of RSV immunisation products' dashboard 34](#_Toc192278549)

[References 36](#_Toc192278550)

# Supplement 1: Search strategies

| 1 | exp Human respiratory syncytial virus/ |
| --- | --- |
| 2 | (Human respiratory syncytial virus or respiratory syncytial virus or RSV).ti,ab,kf. |
| 3 | 1 or 2 |
| 4 | exp respiratory syncytial virus vaccine/ |
| 5 | exp vaccine/ |
| 6 | exp vaccination/ or exp immunization/ |
| 7 | (vaccin* or innoculat$ or jab or immuni#tion* or Nirsevimab or Monoclonal antibod* or PF?06928316 or RSVpreF or Prophyla* or Arexvy or Abrysvo or Mresvia).mp. |
| 8 | 4 or 5 or 6 or 7 |
| 9 | exp drug safety/ |
| 10 | exp adverse event/ or exp drug monitoring/ or exp adverse drug reaction/ |
| 11 | ((adverse or side) adj2 (effect* or reaction* or event*)).mp. |
| 12 | (safety or harm* or disadvantage*).mp. |
| 13 | exp clinical effectiveness/ |
| 14 | exp drug efficacy/ |
| 15 | (effect* or efficac* or benefit* or advantage*).mp. |
| 16 | 9 or 10 or 11 or 12 or 13 or 14 or 15 |
| 17 | 3 and 8 and 16 |
| 18 | 17 not ((exp animal/ or nonhuman/) not exp human/) |
| 19 | 18 and (202212* or 2023* or 2024*).dd. |

Table S1. Search strategy for Ovid Embase (Embase Classic+Embase <1947 to 2025 February 05>)

| 1 | exp Respiratory Syncytial Virus, Human/ |
| --- | --- |
| 2 | (Human respiratory syncytial virus or respiratory syncytial virus or RSV).ti,ab,kf. |
| 3 | 1 or 2 |
| 4 | Viral Vaccines/ |
| 5 | exp Immunization/ |
| 6 | (vaccin* or innoculat$ or jab or immuni#tion* or Nirsevimab or Monoclonal antibod* or PF?06928316 or RSVpreF or Prophyla* or Arexvy or Abrysvo or Mresvia).mp. |
| 7 | 4 or 5 or 6 |
| 8 | exp "Drug-Related Side Effects and Adverse Reactions"/ or exp Adverse Drug Reaction Reporting Systems/ |
| 9 | exp Safety/ |
| 10 | ((adverse or side) adj2 (effect* or reaction* or event*)).mp. |
| 11 | (safety or harm* or disadvantage*).mp. |
| 12 | Comparative Effectiveness Research/ |
| 13 | exp Vaccine Efficacy/ |
| 14 | (effect* or efficac* or benefit* or advantage*).mp. |
| 15 | 8 or 9 or 10 or 11 or 12 or 13 or 14 |
| 16 | 3 and 7 and 15 |
| 17 | exp animals/ not humans.sh. |
| 18 | 16 not 17 |
| 19 | 18 and (202212* or 2023* or 2024*).ed. |

Table S2. Search strategy for Ovid Medline (Ovid MEDLINE(R) ALL <1946 to February 05, 2025>)

| 1 | (Human respiratory syncytial virus or respiratory syncytial virus or RSV).ti,ab,hw. |
| --- | --- |
| 2 | exp vaccines/ |
| 3 | exp immunization/ |
| 4 | (vaccin* or innoculat$ or jab or immuni#tion* or Nirsevimab or Monoclonal antibod* or PF?06928316 or RSVpreF or Prophyla* or Arexvy or Abrysvo or Mresvia).mp. |
| 5 | 2 or 3 or 4 |
| 6 | exp adverse effects/ |
| 7 | ((adverse or side) adj2 (effect* or reaction* or event*)).mp. |
| 8 | (safety or harm* or disadvantage*).mp. |
| 9 | exp efficacy/ |
| 10 | (effectiveness or efficac* or benefit* or advantage*).mp. |
| 11 | 6 or 7 or 8 or 9 or 10 |
| 12 | 1 and 5 and 11 |
| 13 | 12 and (202212* or 2023* or 2024*).dp. |

Table S3. Search strategy for Global Health database (<1973 to 2025 Week 6>)

# Supplement 2: Excluded studies

Table S4. Studies excluded after full-text screening with reasons for exclusion

| **Reason** | **Study** |
| --- | --- |
| No full text available (e.g. abstracts, editorials) | 1. Blauvelt et al. (2024)^1^ 2. Georgiadis et al. (2024)^2^ 3. Hamid et al. (2024)^3^ 4. Hsiao et al. (2024)^4^ 5. La et al. (2024)^5^ 6. Lai et al. (2023)^6^ 7. Loeb et al. (2024)^7^ 8. Lorenzini et al. (2023)^8^ 9. Martin et al. (2024)^9^ 10. Molnar et al. (2023)^10^ 11. Rallabhandi et al. (2024)^11^ |
| No real-world evidence | 1. Adhikari et al. (2024)^12^ 2. Brault et al. (2024)^13^ 3. Du et al. (2025)^14^ 4. Hansen et al. (2024)^15^ 5. Maculaitis et al. (2024)^16^ 6. Mazagatos et al. (2024)^17^ 7. Sallam et al. (2025)^18^ 8. Trubin et al. (2024)^19^ |
| No uptake data | 1. Alami et al. (2024)^20^ 2. Carcione et al. (2025)^21^ 3. Domnich et al. (2025)^22^ 4. Falsey et al. (2024)^23^ 5. Perramon-Malavez et al. (2024)^24^ 6. Mestre-Ferrandiz et al. (2024)^25^ |
| No data on nirsevimab or approved RSV vaccines | 1. Bracaloni et al. (2024)^26^ 2. Grahic-Mujcinovic et al. (2024)^27^ 3. Moro et al. (2024)^28^ 4. Raguz et al. (2022)^29^ 5. Remmele et al. (2024)^30^ |
| Duplicates (studies included or excluded in a previous months’ search) | 1. Levy et al. (2024)^31^ 2. Lopez-Lacort et al. (2025)^32^ 3. Moline et al. (2025)^33^ 4. Rodriguez-Fernandez et al. (2024)^34^ |
| Clinical trial | 1. Biegus et al. (2024)^35^ 2. Domachowske et al. (2022)^36^ 3. Domachowske et al. (2023)^37^ |
| Not in English | 1. Novoa Pizarro et al. (2023)^38^ 2. Rodriguez-Fernandez et al. (2024)^34^ |

# Supplement 3: ‘Risk of bias’ quality assessment

To assess the risk of bias, we used Joanna Briggs Institute (JBI) Critical Appraisal Tools for cohort studies, case control studies, cross-sectional studies, or quasi-experimental studies depending on the study designs.^39^

‘Risk of bias’ assessments were completed by two reviewers for each study, marking each criterion as ‘yes’, ‘no’, ‘unclear’, or ‘not applicable’. Conflicts were resolved in a discussion. The number of ‘yes’ responses for each study (total) were divided by the total number of criteria to obtain a score ranging from 0% to 100%. Studies with scores 50% and less were classified as ‘high risk’, scores between 51% and 75% were considered ‘medium risk’, and studies with scores above 75% were considered ‘low risk’.

Sensitivity analyses of the pooled estimates for uptake of nirsevimab (in Spain, United States, and France) and RSV vaccines for older adults (United States) were conducted using data from studies classified as at ‘low risk of bias’. This sensitivity analysis could not be carried out for the uptake of RSV maternal vaccine (United States) because less than three studies reporting on this product were classified as at ‘low risk of bias’.

**Table S5. ‘Risk of bias’ assessment for cohort studies**

**JBI criteria:**

Q1: Were the two groups similar and recruited from the same population?

Q2: Were the exposures measured similarly to assign people to both exposed and unexposed groups?

Q3: Was the exposure measured in a valid and reliable way?

Q4: Were confounding factors identified?

Q5: Were strategies to deal with confounding factors stated?

Q6: Were the groups/participants free of the outcome at the start of the study (or at the moment of exposure)?

Q7: Were the outcomes measured in a valid and reliable way?

Q8: Was the follow up time reported and sufficient to be long enough for outcomes to occur?

Q9: Was follow up complete, and if not, were the reasons to loss to follow up described and explored?

Q10: Were strategies to address incomplete follow up utilized?

Q11: Was appropriate statistical analysis used?

| **Study** | **Q1** | **Q2** | **Q3** | **Q4** | **Q5** | **Q6** | **Q7** | **Q8** | **Q9** | **Q10** | **Q11** | **Total** | **Total (%)** | **Risk of bias assessment** |
| --- | --- | --- | --- | --- | --- | --- | --- | --- | --- | --- | --- | --- | --- | --- |
| Ares-Gomez et al. (2024)^40^ | Yes | Yes | Yes | Yes | Yes | Unclear | Yes | Unclear | Yes | Not applicable | Yes | 8 | 72.7% | **medium** |
| Barbas Del Buey et al. (2024)^41^ | Yes | Yes | Yes | Yes | Yes | Unclear | Yes | Yes | Yes | Not applicable | Yes | 9 | 81.8% | **low** |
| Birabaharan et al. (2024)^42^ | Yes | Yes | Yes | Yes | Yes | Yes | Yes | Yes | Yes | Yes | Yes | 11 | 100.0% | **low** |
| Coma et al. (2024)^43^ | Yes | Yes | Yes | Yes | Yes | Yes | Yes | Unclear | Yes | Not applicable | Yes | 9 | 81.8% | **low** |
| Estrella-Porter et al. (2024)^44^ | Yes | Yes | Yes | Yes | Yes | No | Yes | Unclear | Yes | Not applicable | Yes | 8 | 72.7% | **medium** |
| Ezpeleta et al. (2024)^45^ | Yes | Yes | Yes | Yes | Yes | Yes | Yes | No | Yes | Yes | Yes | 10 | 90.9% | **low** |
| Homo et al. (2024)^46^ | Yes | Yes | Unclear | No | No | Yes | Yes | Unclear | Unclear | Unclear | No | 4 | 36.36% | **high** |
| Jimeno Ruiz et al. (2024)^47^ | Yes | Yes | Yes | Yes | Yes | Unclear | Yes | Unclear | Unclear | Unclear | Yes | 7 | 63.6% | **medium** |
| Mallah et al. (2024)^48^ | Yes | Yes | Yes | Yes | Yes | Yes | Yes | Unclear | Yes | Not applicable | Yes | 9 | 81.8% | **low** |
| Martinon-Torres et al. (2024)^49^ | Yes | Yes | Yes | Unclear | No | Yes | Yes | Unclear | No | No | No | 5 | 45.5% | **high** |
| Puckett et al. (2025)^50^ | Yes | Yes | Yes | Yes | Yes | Yes | Yes | Yes | Yes | Unclear | Yes | 10 | 90.9% | **low** |
| Reses et al. (2023)^51^ | Yes | Yes | Yes | No | No | No | Yes | Unclear | No | No | Yes | 5 | 45.5% | **high** |
| Reses et al. (2024)^52^ | Yes | Yes | Yes | No | No | No | Yes | Unclear | No | No | Yes | 5 | 45.5% | **high** |
| Son et al. (2024)^53^ | Yes | Yes | Yes | Yes | Yes | Unclear | Yes | Yes | Unclear | No | Yes | 8 | 72.7% | **medium** |

**Table S6. ‘Risk of bias’ assessment for cross-sectional studies**

**JBI criteria:**

Q1: Were the criteria for inclusion in the sample clearly defined?

Q2: Were the study subjects and the setting described in detail?

Q3: Was the exposure measured in a valid and reliable way?

Q4: Were objective, standard criteria used for measurement of the condition?

Q5: Were confounding factors identified?

Q6: Were strategies to deal with confounding factors stated?

Q7: Were the outcomes measured in a valid and reliable way?

Q8: Was appropriate statistical analysis used?

| **Study** | **Q1** | **Q2** | **Q3** | **Q4** | **Q5** | **Q6** | **Q7** | **Q8** | **Total** | **Total (%)** | **Risk of bias assessment** |
| --- | --- | --- | --- | --- | --- | --- | --- | --- | --- | --- | --- |
| Geng et al. (2024)^54^ | Yes | Yes | No | Unclear | Yes | Yes | Yes | Yes | 6 | 75.0% | **medium** |
| Kemp et al. (2025)^55^ | Yes | Yes | Yes | Unclear | No | Not applicable | Yes | Yes | 5 | 62.5% | **medium** |
| Motta et al. (2025)^56^ | Yes | Unclear | No | Unclear | Yes | Yes | No | Yes | 4 | 50.0% | **high** |
| Pérez Martín and Zornoza Moreno (2024)^57^ | Yes | Yes | Yes | Yes | Unclear | No | Yes | Yes | 6 | 75.0% | **medium** |
| Razzaghi et al. (2024)^58^ | Yes | Yes | No | Yes | No | No | Yes | No | 4 | 50.0% | **high** |

**Table S7. ‘Risk of bias’ assessment for case-control studies**

**JBI criteria:**

Q1: Were the groups comparable other than the presence of disease in cases or the absence of disease in controls?

Q2: Were cases and controls matched appropriately?

Q3: Were the same criteria used for identification of cases and controls?

Q4: Was exposure measured in a standard, valid and reliable way?

Q5: Was exposure measured in the same way for cases and controls?

Q6: Were confounding factors identified?

Q7: Were strategies to deal with confounding factors stated?

Q8: Were outcomes assessed in a standard, valid and reliable way for cases and controls?

Q9: Was the exposure period of interest long enough to be meaningful?

Q10: Was appropriate statistical analysis used?

| **Study** | **Q1** | **Q2** | **Q3** | **Q4** | **Q5** | **Q6** | **Q7** | **Q8** | **Q9** | **Q10** | **Total** | **Total (%)** | **Risk of bias assessment** |
| --- | --- | --- | --- | --- | --- | --- | --- | --- | --- | --- | --- | --- | --- |
| Aguera et al. (2024)^59^ | Yes | No | Yes | Yes | Yes | Yes | Yes | Yes | Unclear | Yes | 8 | 80.0% | **low** |
| Assad et al. (2024)^60^ | Yes | Yes | Yes | Yes | Yes | Yes | Yes | Yes | Yes | Yes | 10 | 100.0% | **low** |
| Carbajal et al. (2024)^61^ | Yes | No | Yes | Yes | Yes | Yes | Yes | Yes | Unclear | Yes | 8 | 80.0% | **low** |
| Lefferts et al. (2024)^62^ | Yes | Unclear | Yes | Yes | Yes | Yes | Yes | Yes | Yes | Yes | 9 | 90.0% | **low** |
| Lenglart et al. (2025)^63^ | Yes | Yes | Yes | Yes | Yes | Yes | Yes | Unclear | Unclear | Yes | 8 | 80.0% | **low** |
| López-Lacort et al. (2024)^64^ | Yes | No | Yes | Yes | Yes | Unclear | Unclear | Yes | Unclear | Unclear | 5 | 50.0% | **high** |
| López-Lacort et al. (2025)^32^ | Yes | Yes | Yes | Yes | Yes | Unclear | Unclear | Yes | Unclear | Yes | 7 | 70.0% | **medium** |
| Moline et al. (2024a)^65^ | Yes | No | Yes | Yes | Yes | Yes | Yes | Yes | Yes | Yes | 9 | 90.0% | **low** |
| Moline et al. (2024b)^33^ | Yes | No | Yes | Yes | Yes | Yes | Yes | Yes | Unclear | Yes | 8 | 80.0% | **low** |
| Paireau et al. (2024)^66^ | Yes | No | Yes | Yes | Yes | Yes | Yes | Yes | Yes | Yes | 9 | 90.0% | **low** |
| Payne et al. (2024)^67^ | Yes | No | Yes | Yes | Yes | Yes | Yes | Yes | Yes | Yes | 9 | 90.0% | **low** |
| Surie et al. (2024)^68^ | Yes | Yes | Yes | Yes | Yes | Yes | Yes | Yes | Unclear | Yes | 9 | 90.0% | **low** |
| Tartof et al. (2024)^69^ | Yes | No | Yes | Yes | Yes | Yes | Yes | Yes | Unclear | Yes | 8 | 80.0% | **low** |
| Xu et al. (2024)^70^ | Yes | Unclear | Yes | Yes | Yes | Yes | Yes | Yes | Yes | Yes | 9 | 90.0% | **low** |

**Table S8. ‘Risk of bias’ assessment for quasi-experimental (incl. interrupted time series) studies**

**JBI criteria:**

Q1: Is it clear in the study what is the “cause” and what is the “effect” (i.e. there is no confusion about which variable comes first)?

Q2: Was there a control group?

Q3: Were participants included in any comparisons similar?

Q4: Were the participants included in any comparisons receiving similar treatment/care, other than the exposure or intervention of interest?

Q5: Were there multiple measurements of the outcome, both pre and post the intervention/exposure?

Q6: Were the outcomes of participants included in any comparisons measured in the same way?

Q7: Were outcomes measured in a reliable way?

Q8: Was follow-up complete and if not, were differences between groups in terms of their follow-up adequately described and analyzed?

Q9: Was appropriate statistical analysis used?

| **Study** | **Q1** | **Q2** | **Q3** | **Q4** | **Q5** | **Q6** | **Q7** | **Q8** | **Q9** | **Total** | **Total (%)** | **Risk of bias assessment** |
| --- | --- | --- | --- | --- | --- | --- | --- | --- | --- | --- | --- | --- |
| Alejandre et al. (2024)^71^ | Yes | Unclear | Yes | Yes | No | Yes | Yes | Yes | Yes | 7 | 77.8% | low |
| Andina Martinez et al. (2024)^72^ | Yes | Unclear | Unclear | Yes | Yes | Yes | Yes | Yes | Yes | 7 | 77.78% | low |
| Cantais et al. (2024)^73^ | Yes | Unclear | Yes | Unclear | No | Yes | Unclear | Unclear | Unclear | 3 | 33.3% | high |
| Chauvel et al. (2024)^74^ | Yes | Yes | Yes | Yes | Yes | Yes | Yes | Unclear | Yes | 8 | 88.89% | low |
| Consolati et al. (2024)^75^ | Yes | Yes | Unclear | Yes | No | Yes | Yes | No | Yes | 6 | 66.67% | medium |
| Ernst et al. (2024)^76^ | Yes | Unclear | Unclear | Unclear | Yes | Yes | Yes | Yes | Yes | 6 | 66.7% | medium |
| Espeleta-Fox et al. (2024)^77^ | Yes | Unclear | Unclear | Unclear | No | Yes | Unclear | Yes | Unclear | 3 | 33.3% | high |
| Levy et al. (2024)^31^ | Yes | Yes | Unclear | Unclear | Yes | Yes | Yes | Unclear | Yes | 6 | 66.7% | medium |
| Molina Gutierrez et al. (2024)^78^ | Yes | Yes | Yes | Yes | No | Yes | Yes | Unclear | No | 6 | 66.7% | medium |
| Perramon-Malavez et al. (2025)^79^ | Yes | Unclear | Yes | Unclear | No | Yes | Yes | Unclear | Yes | 5 | 55.6% | medium |

# Supplement 4: Sensitivity analysis

Sensitivity analyses were carried out using data from population-based studies classified as ‘low risk of bias’ in the risk of bias assessment (Supplement 3).

Figure S1. Sensitivity analyses of uptake of nirsevimab among eligible children in Spain during the 2023/24 RSV season


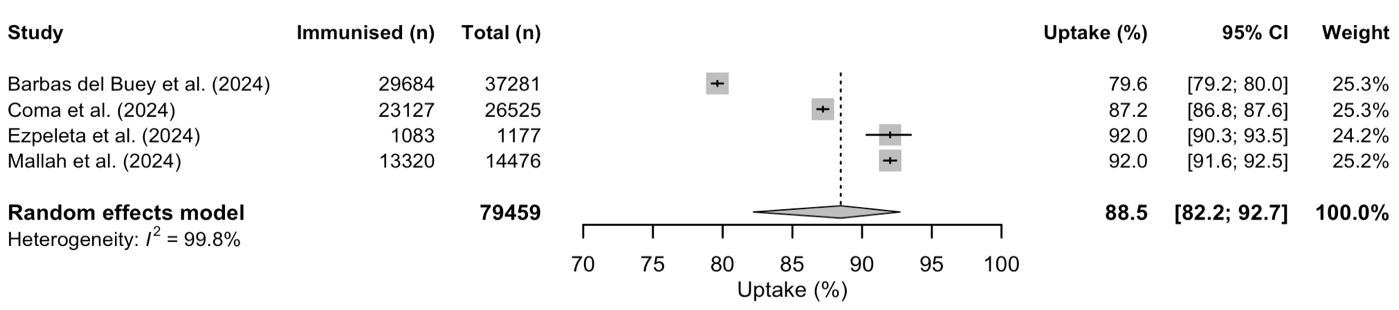


# Supplement 5: Uptake of RSV immunisation products stratified by health status at data collection

For countries and RSV immunisation products with at least three data points, we conducted meta-analysis to compare uptake between subgroups:

(1) individuals with medically attended laboratory-tested RSV-associated acute respiratory infections (ARI) at the time of data collection;

(2) individuals with medically attended all-cause ARI at the time of data collection;

(3) individuals without ARI at the time of data collection.

Figure S2. Uptake (%) of nirsevimab among eligible children in Spain during the 2023/24 RSV season stratified by health status at data collection


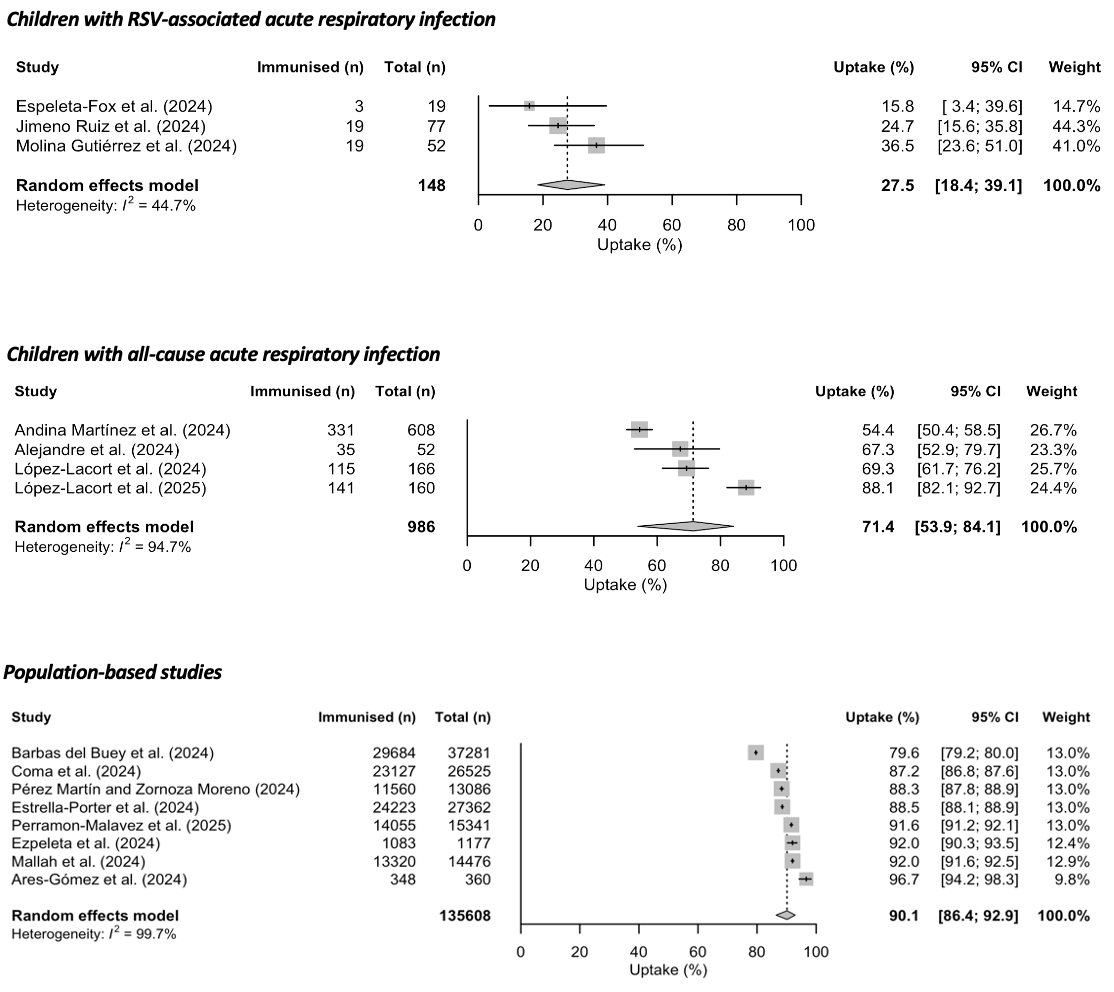


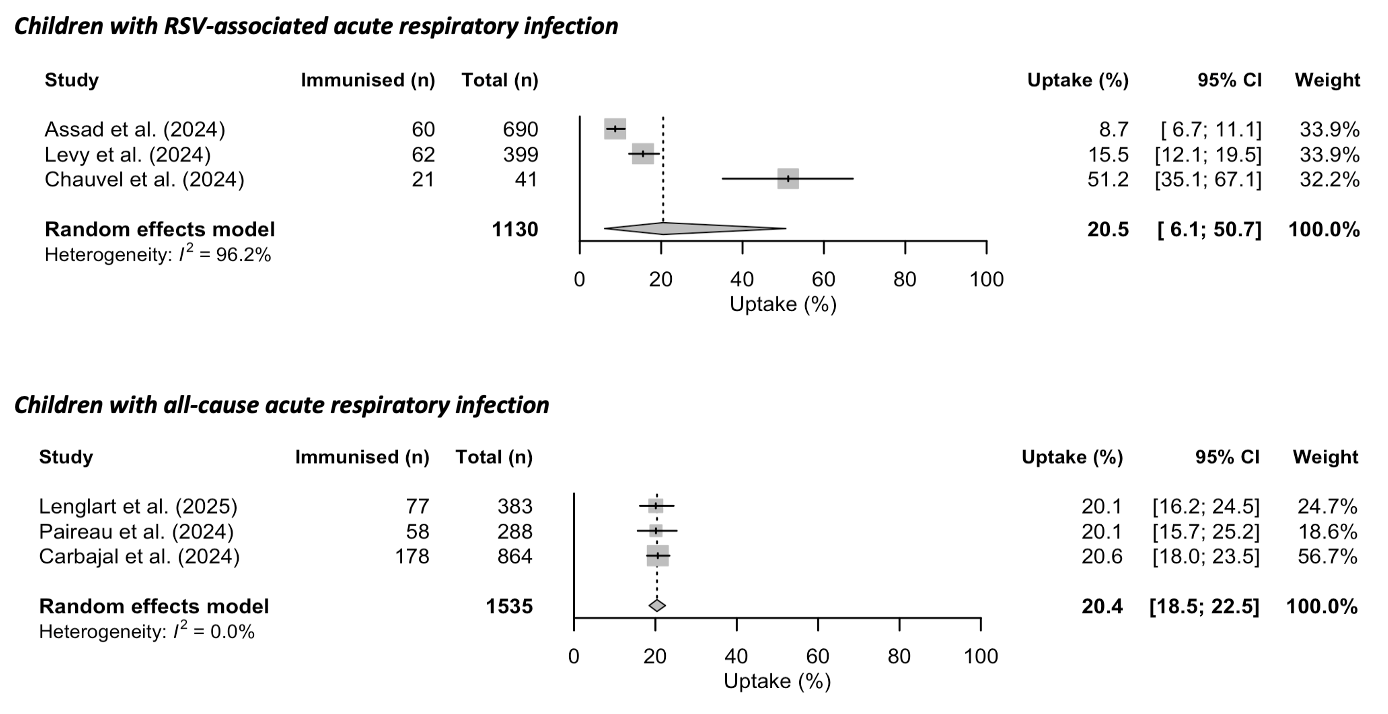
Figure S3. Uptake (%) of nirsevimab among eligible children in France during the 2023/24 RSV season stratified by health status at data collection

Figure S4. Uptake (%) of nirsevimab among eligible children in the United States during the 2023/24 RSV season stratified by health status at data collection


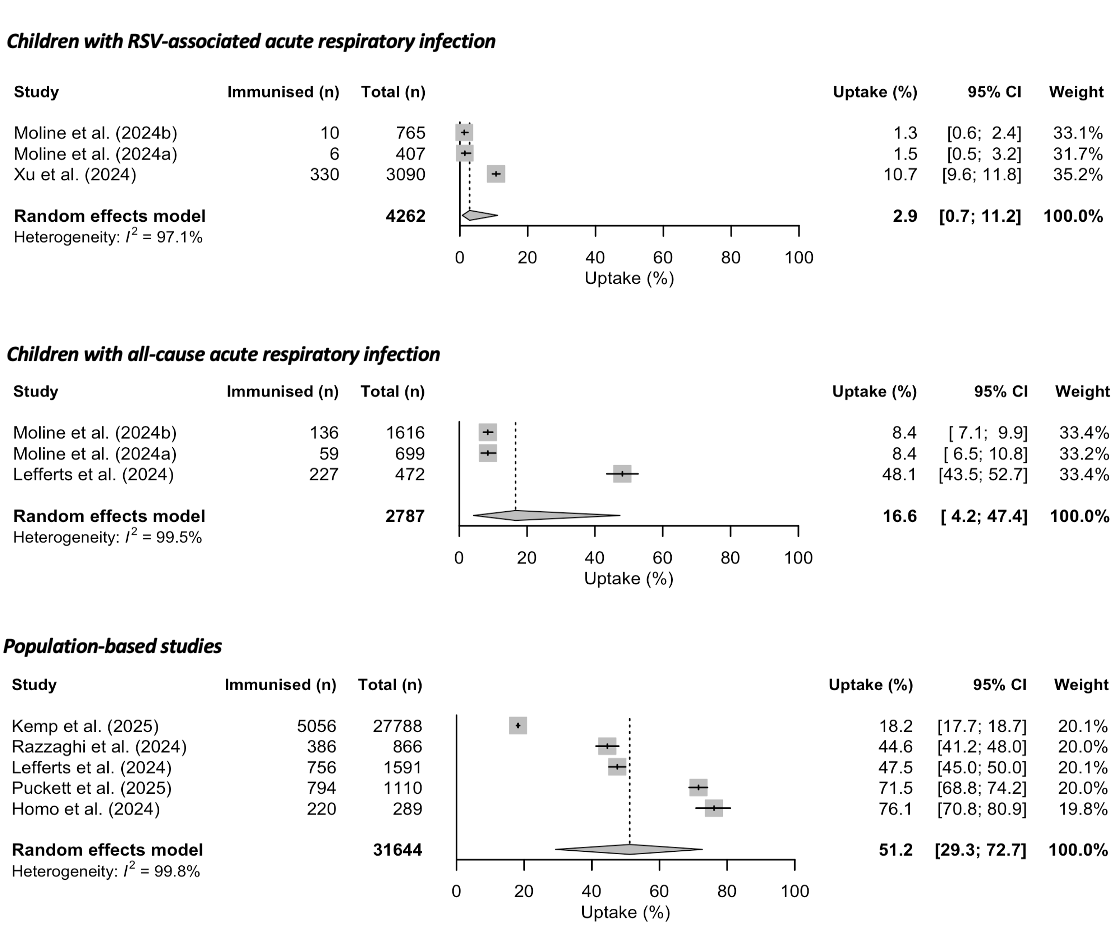


Figure S5. Uptake (%) of RSV maternal vaccination among parents of children who did not have acute respiratory infections at the time of data collection in the United States during the 2023/24 RSV season


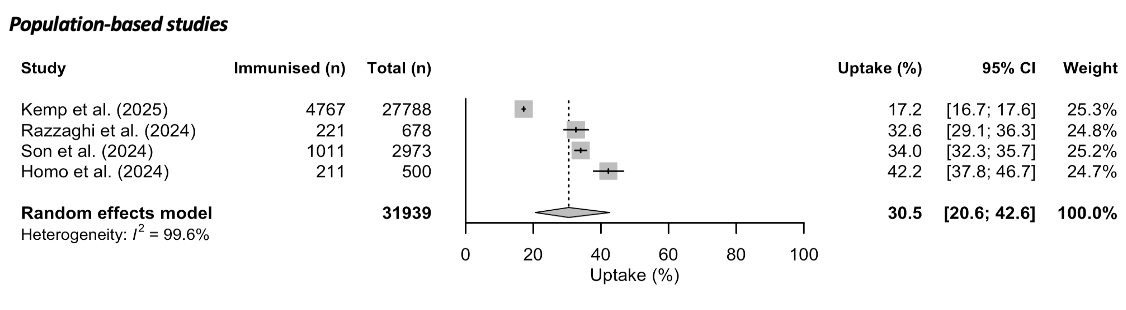


Figure S6. Uptake (%) of RSV vaccines among older adults (≥60 years old) in the United States during the 2023/24 RSV season stratified by their health status at data collection


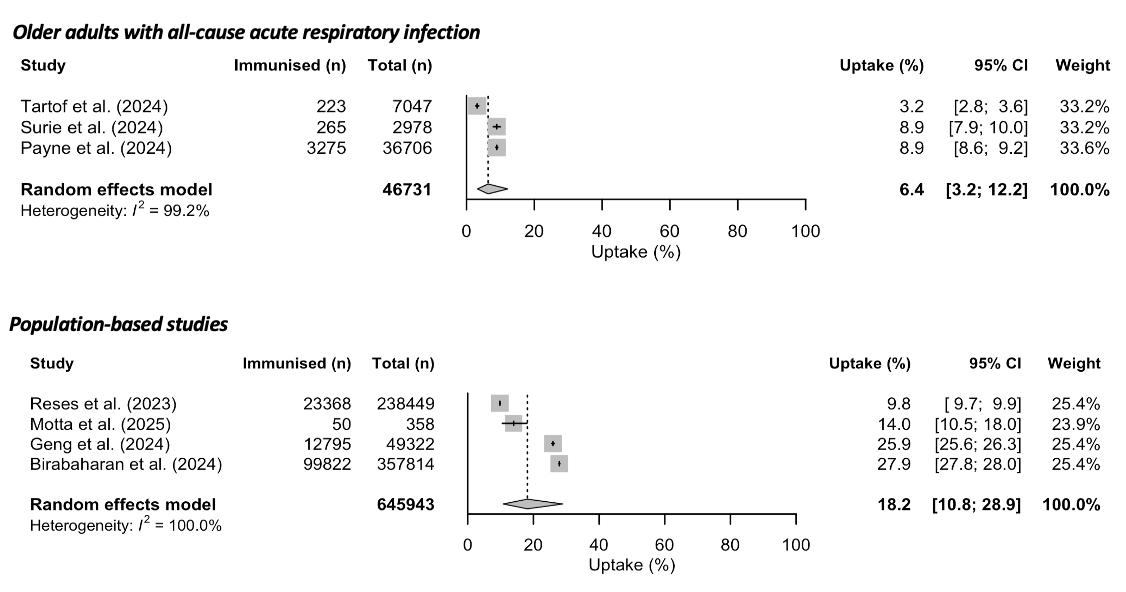


# Supplement 6: Subgroup meta-analysis for data reported in Table 2

## Uptake of nirsevimab in Spain

Figure S7. Uptake of nirsevimab among eligible children in Spain during the 2023/24 RSV season stratified by immunisation programme enrolment group^a^


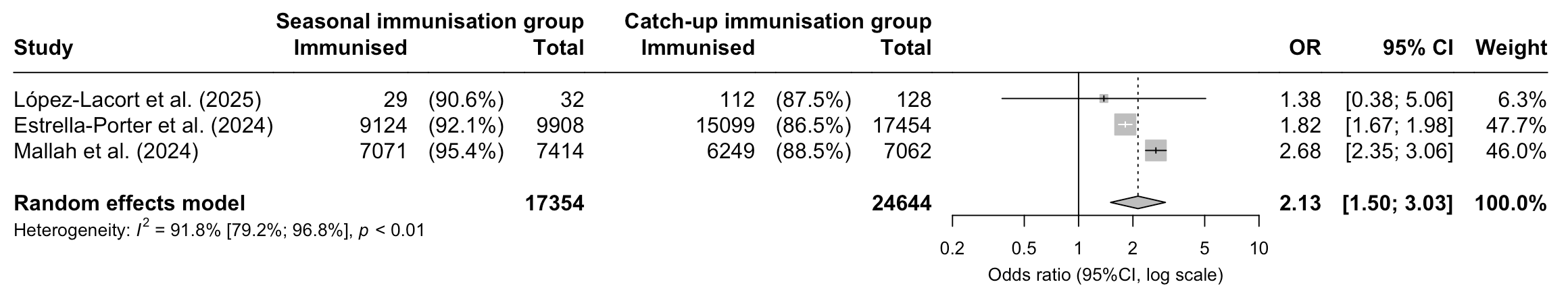


^a^ Seasonal immunisation group: children born after the start of the programme; catch-up immunisation group (ref.): children born within six months before the start of the programme.

Figure S8. Uptake of nirsevimab among eligible children in Spain during the 2023/24 RSV season stratified by sex assigned at birth (males and females (ref.))


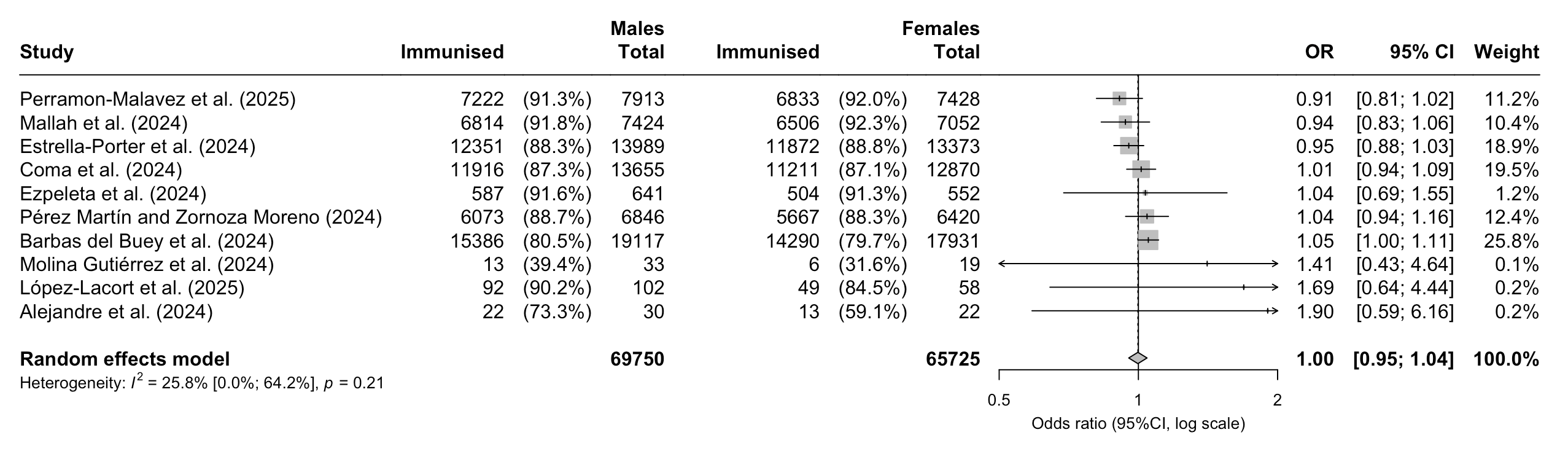


Figure S9. Uptake of nirsevimab among eligible children in Spain during the 2023/24 RSV season stratified by gestational age^a^


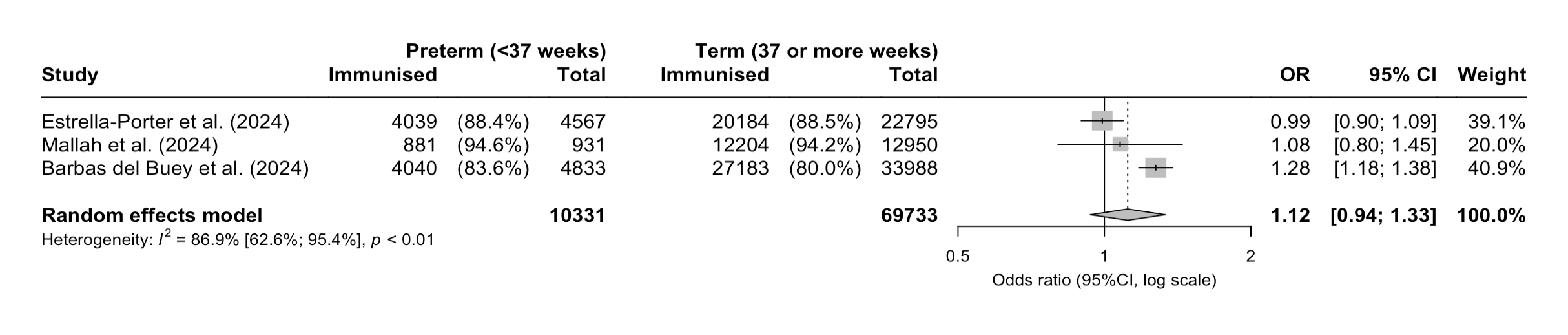


^a^ Preterm: born under 37 weeks of gestation; term: born at 37 or more weeks of gestation (ref.).

Figure S10. Uptake of nirsevimab among eligible children in Spain during the 2023/24 RSV season stratified by nationality^a^


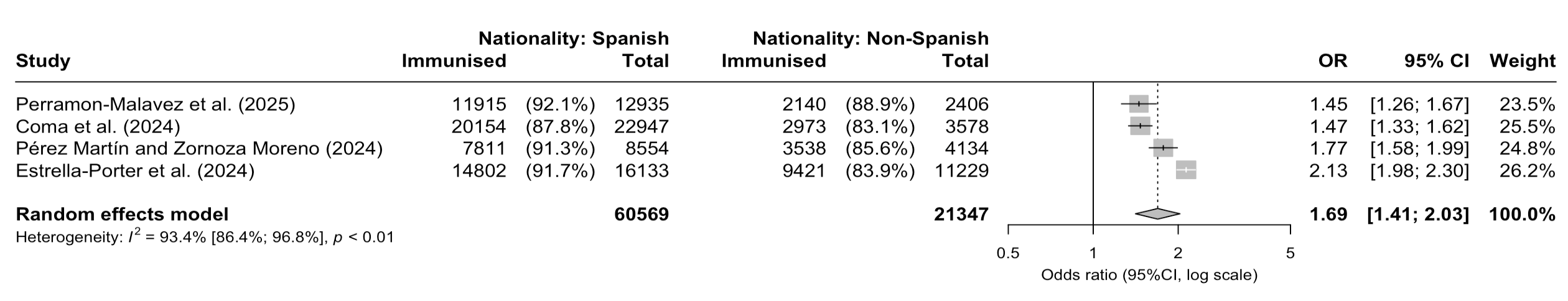


^a^ Children with Spanish nationality compared to children with non-Spanish nationality (ref.).

## Uptake of nirsevimab in France

Figure S11. Uptake of nirsevimab among eligible children in France during the 2023/24 RSV season stratified by age group^a^


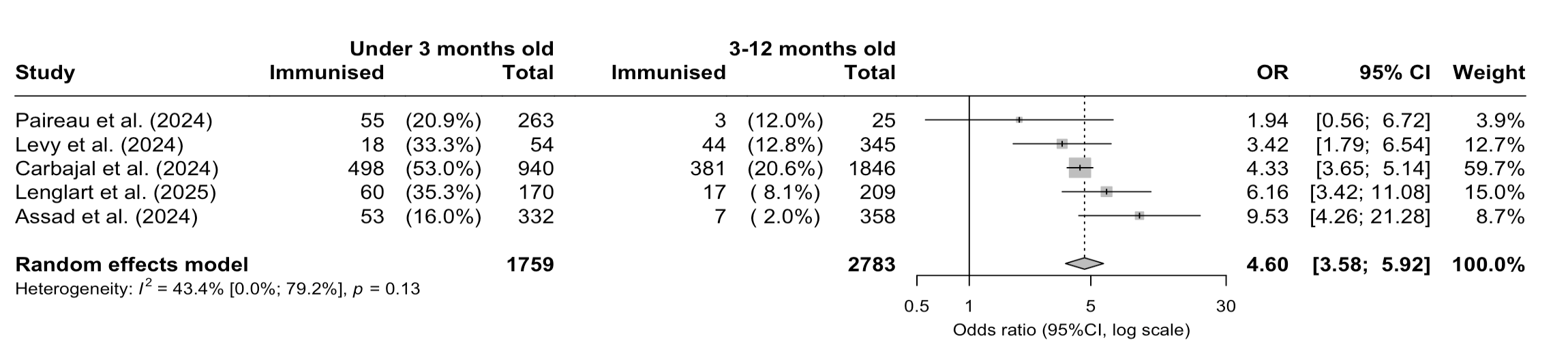


^a^ Children under three months old compared to children aged 3-12 months (ref.).

## Uptake of nirsevimab in the United States

Figure S12. Uptake of nirsevimab among eligible children in the United States during the 2023/24 RSV season stratified by sex assigned at birth (males and females (ref.))


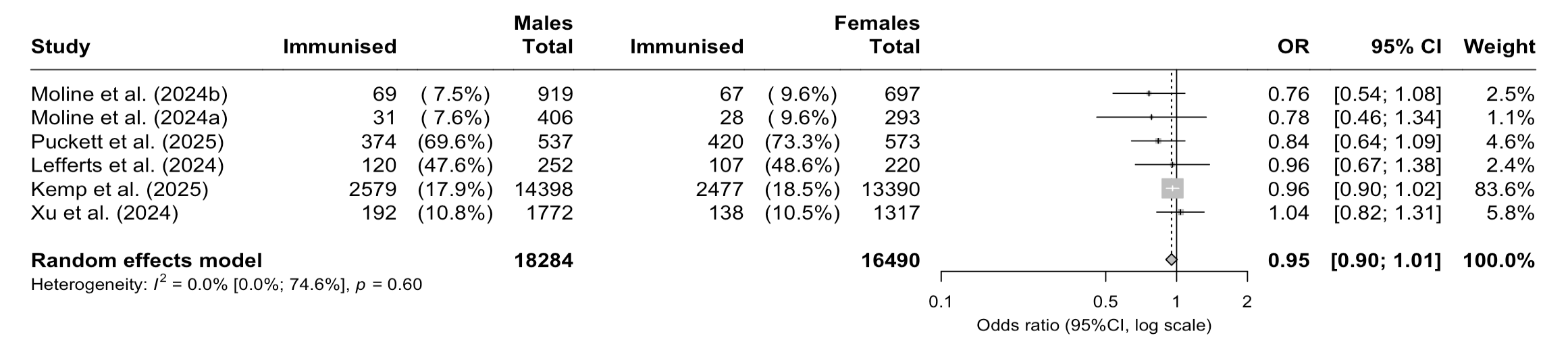


Figure S13. Uptake of nirsevimab among eligible children in the United States during the 2023/24 RSV season stratified by gestational age^a^


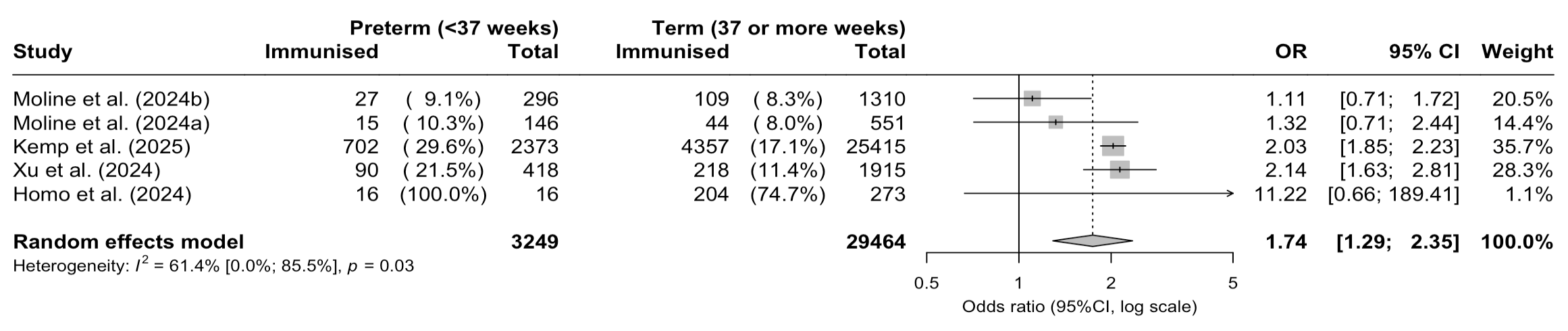


^a^ Preterm: born under 37 weeks of gestation; term: born at 37 or more weeks of gestation (ref.).

Figure S14. Uptake of nirsevimab among eligible children in the United States during the 2023/24 RSV season stratified by presence of comorbidities^a^


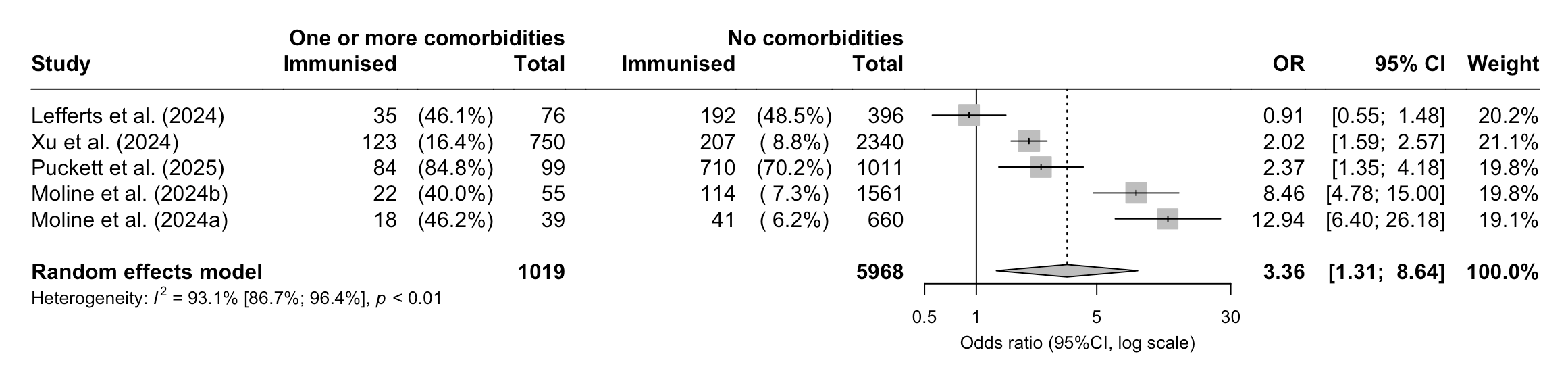


^a^ Children with no comorbidities (ref.) compared to children with one or more comorbidities. In the included studies, comorbidities were defined as congenital heart disease (e.g. abnormalities of aortic arch, hypoplastic left heart syndrome, pulmonary atresia, tricuspid atresia, Tetralogy of Fallot, transposition of the great arteries, partial or total anomalous pulmonary venous return, other abnormalities of heart valves, double outlet right ventricle), immunocompromised status (e.g. transplantation history, leukaemia), cystic fibrosis, Down syndrome, neuromuscular disease (e.g. autonomic dysfunction, instability or dysautonomia, agenesis or hypoplasia of the corpus callosum, muscular dystrophy or spinal muscular atrophy, disorders of tone), pulmonary disease (e.g. reactive airway disease), chronic lung disease of prematurity (e.g. bronchopulmonary dysplasia, bronchiolitis obliterans, chronic respiratory failure with CPAP/BIPAP/ventilator, pulmonary hypertension, interstitial lung disease), prematurity (gestational age under 37 weeks), anaemia, small for gestational age (birth weight <2500 grams).

Figure S15. Uptake of nirsevimab among eligible children in the United States during the 2023/24 RSV season stratified by health insurance type^a^


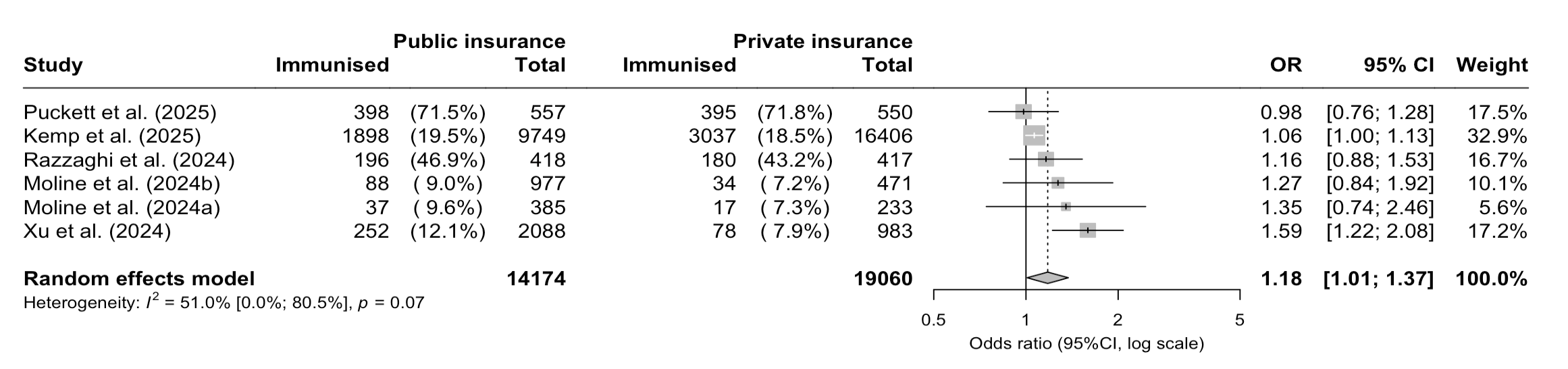


^a^ Private (or military insurance; ref.) compared to public insurance.

Figure S16. Uptake of nirsevimab among eligible children in the United States during the 2023/24 RSV season stratified by health insurance type^a^


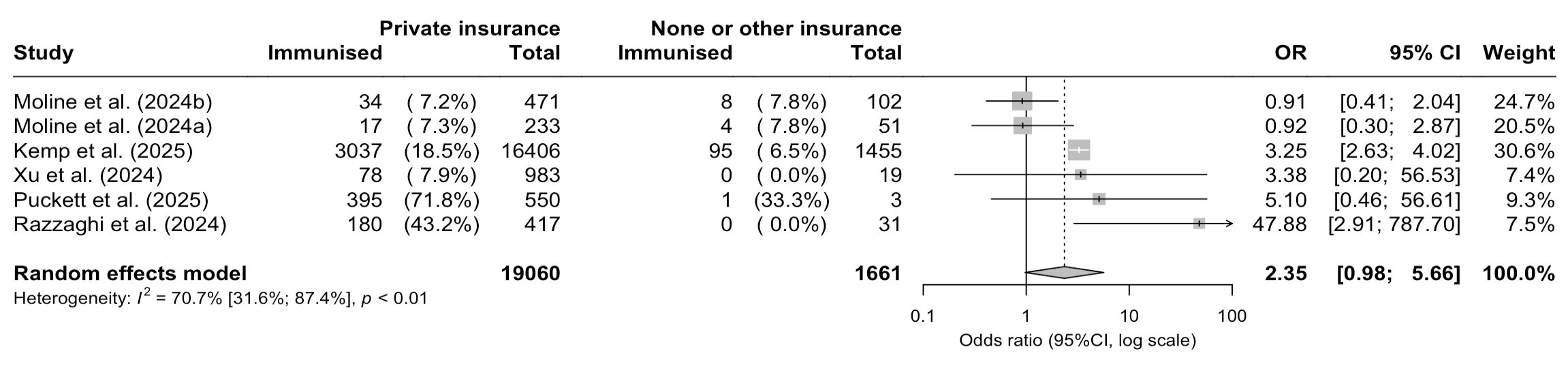
 ^a^ No or other insurance (ref.) compared to private insurance.

Figure S17. Uptake of nirsevimab among eligible children in the United States during the 2023/24 RSV season stratified by health insurance type^a^


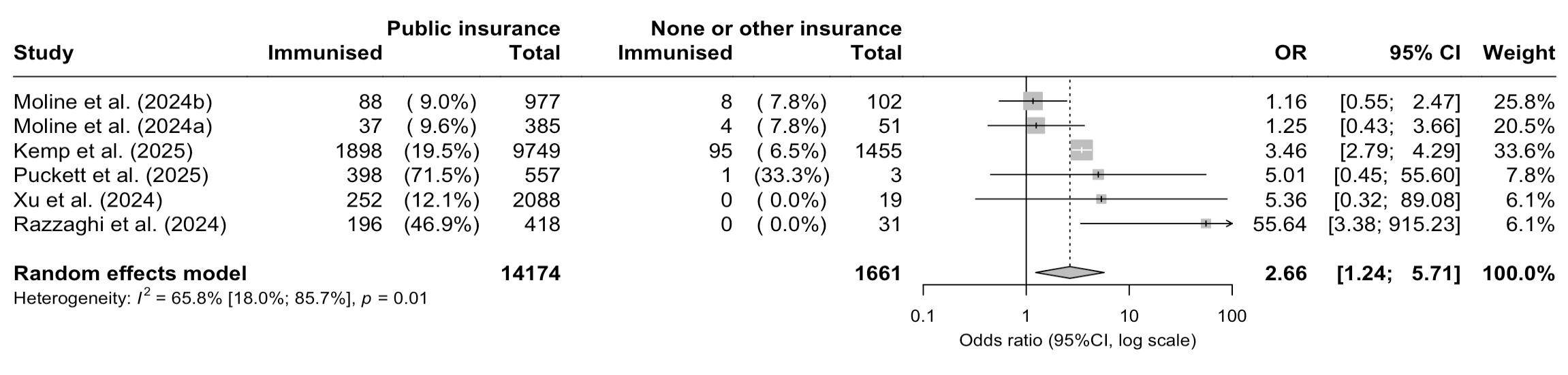
^a^ No or other insurance (ref.) compared to public insurance.

Figure S18. Uptake of nirsevimab among eligible children in the United States during the 2023/24 RSV season stratified by area of residence^a^


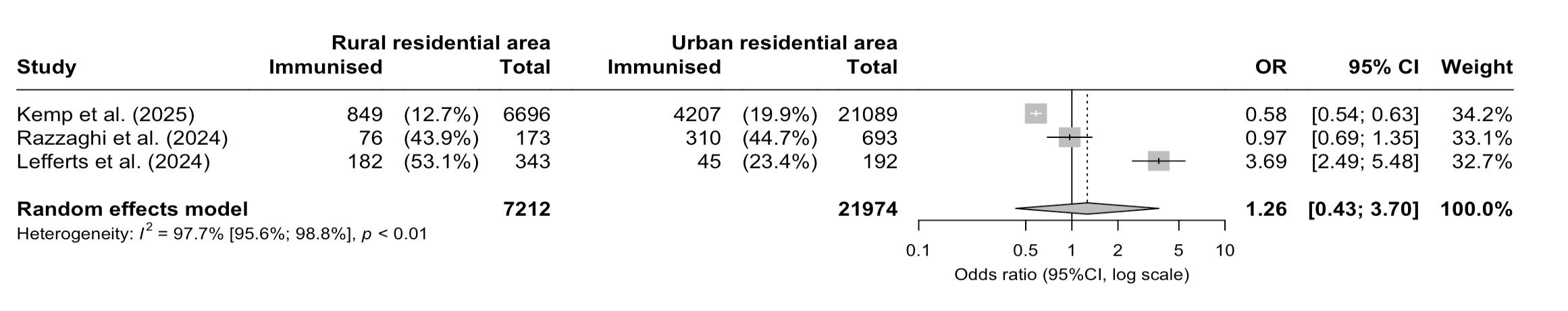


^a^ Urban residential area (ref.) compared to rural residential area.

Figure S19. Uptake of nirsevimab among eligible children in the United States during the 2023/24 RSV season stratified by racial group^a^


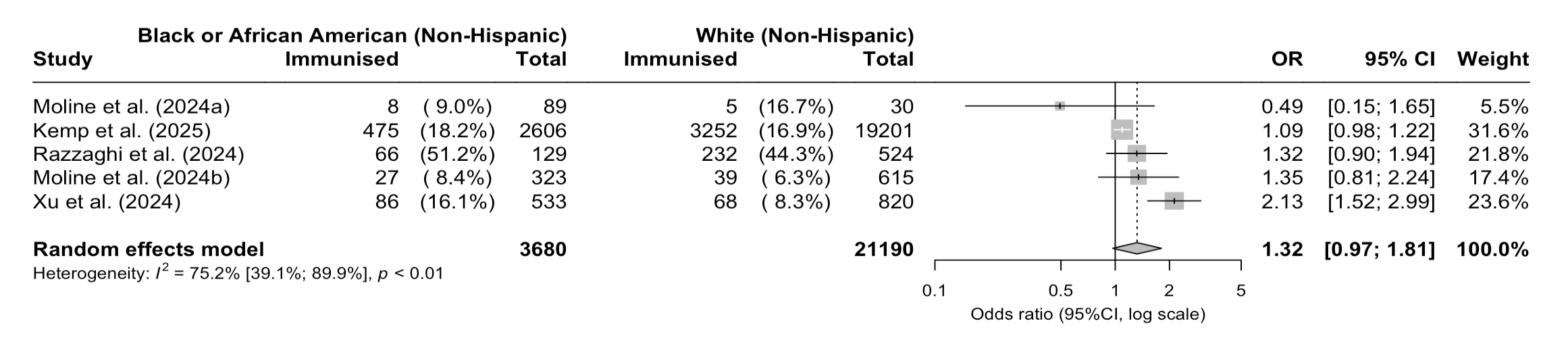


^a^ White (non-Hispanic) population (ref.) compared to Black or African American (non-Hispanic) population.

Figure S20. Uptake of nirsevimab among eligible children in the United States during the 2023/24 RSV season stratified by racial group^a^


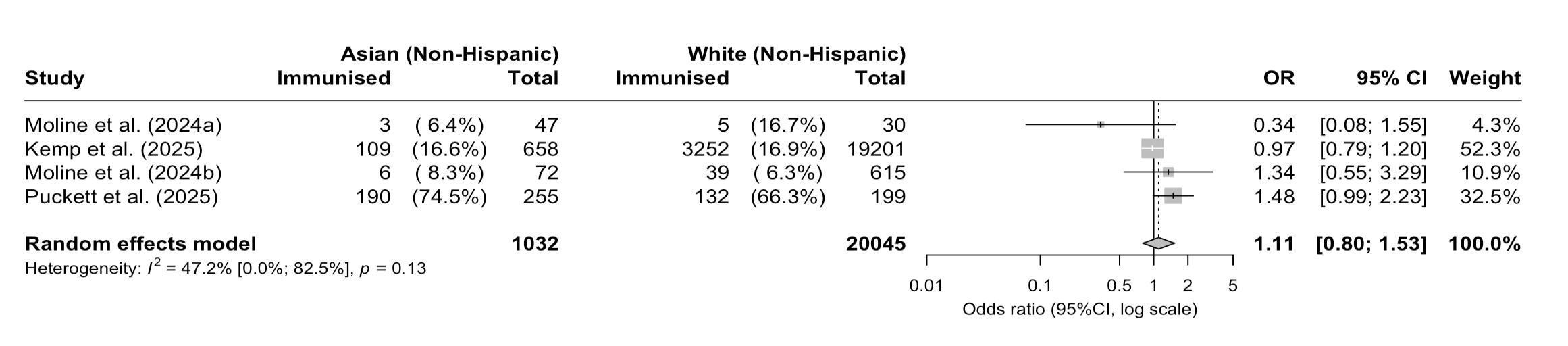


^a^ White (non-Hispanic) population (ref.) compared to Asian (non-Hispanic) population.

Figure S21. Uptake of nirsevimab among eligible children in the United States during the 2023/24 RSV season stratified by racial group^a^


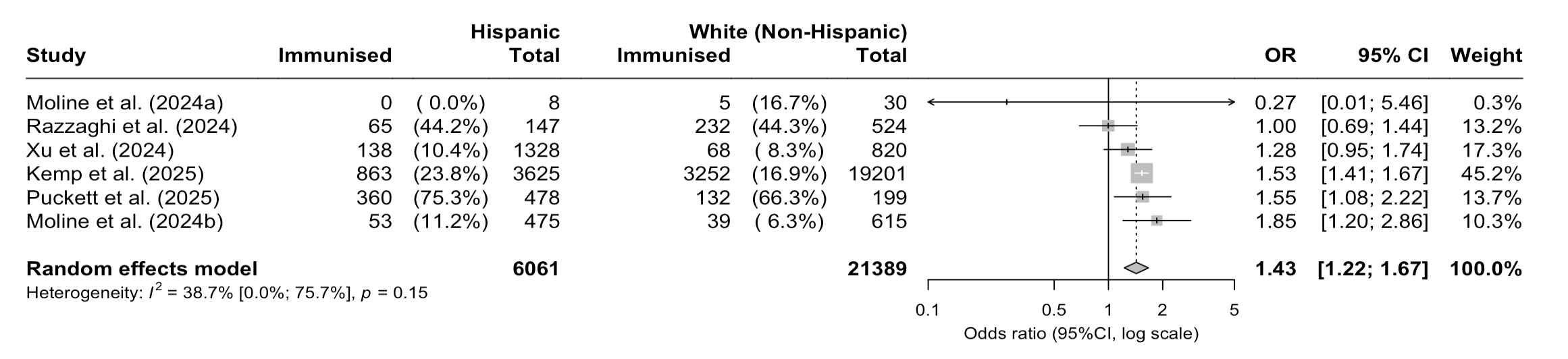


^a^ White (non-Hispanic) population (ref.) compared to Hispanic population.

Figure S22. Uptake of nirsevimab among eligible children in the United States during the 2023/24 RSV season stratified by racial group^a^


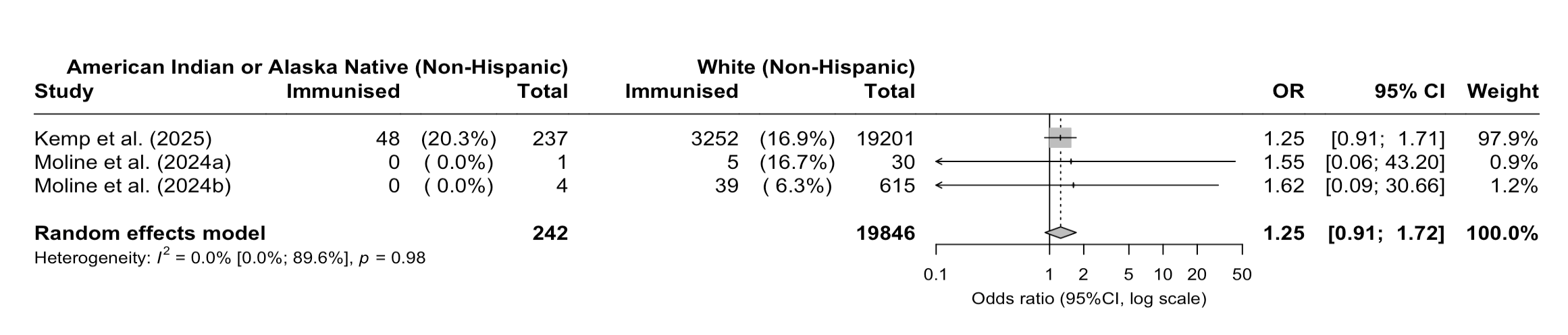


^a^ White (non-Hispanic) population (ref.) compared to American Indian or Alaska Native (non-Hispanic) population.

Figure S23. Uptake of nirsevimab among eligible children in the United States during the 2023/24 RSV season stratified by racial group^a^


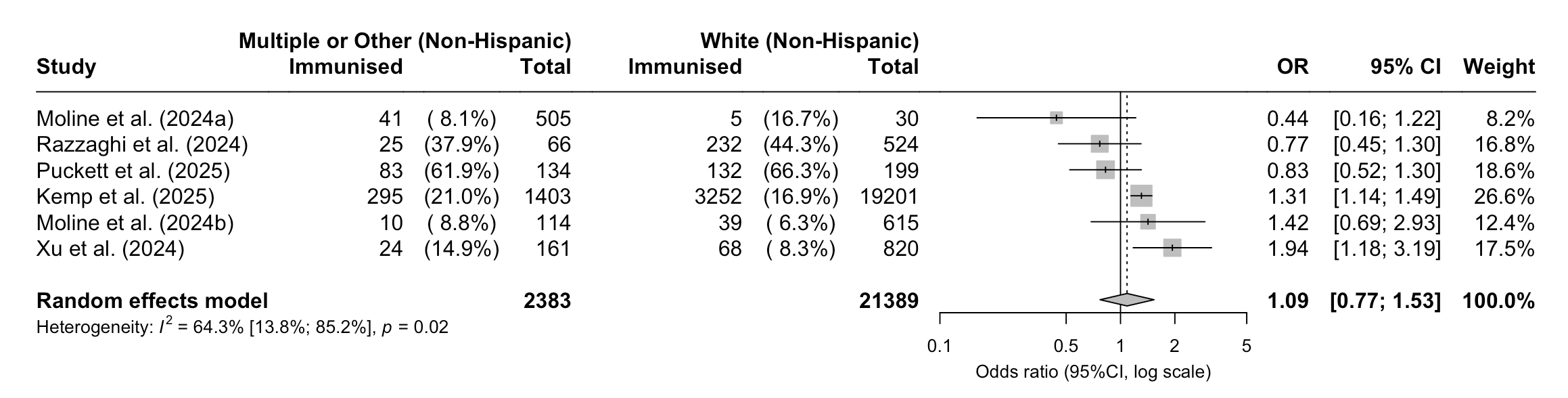


^a^ White (non-Hispanic) population (ref.) compared to population groups defined as multiple or other in the included studies.

Figure S24. Uptake of nirsevimab among eligible children in the United States during the 2023/24 RSV season stratified by ethnic group^a^


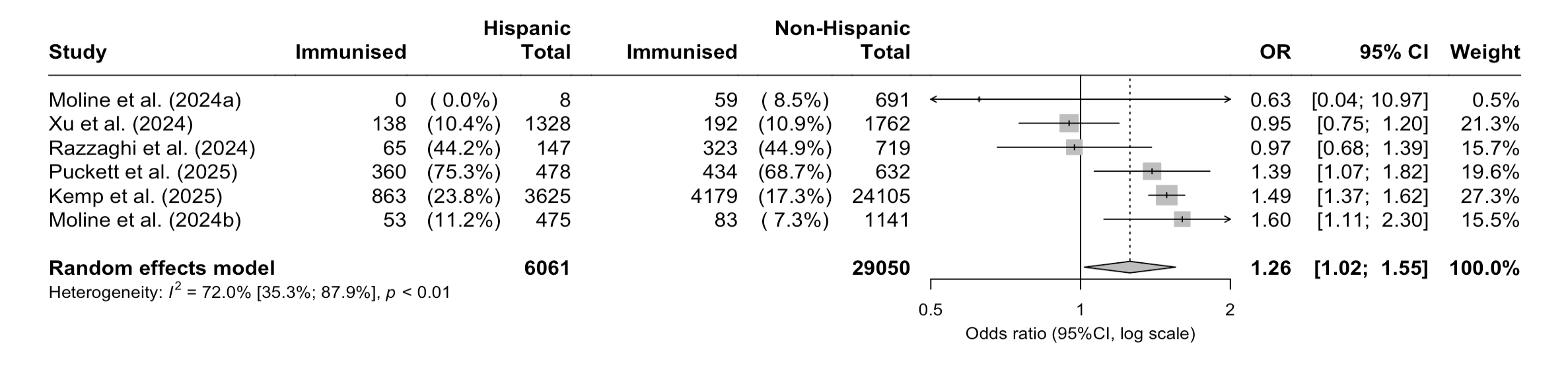


^a^ All non-Hispanic population (ref.) compared to Hispanic population

## Uptake of RSV maternal vaccine in the United States

Figure S25. Uptake of RSV maternal vaccine among eligible parents of children in the United States during the 2023/24 RSV season stratified by infants’ gestational age^a^


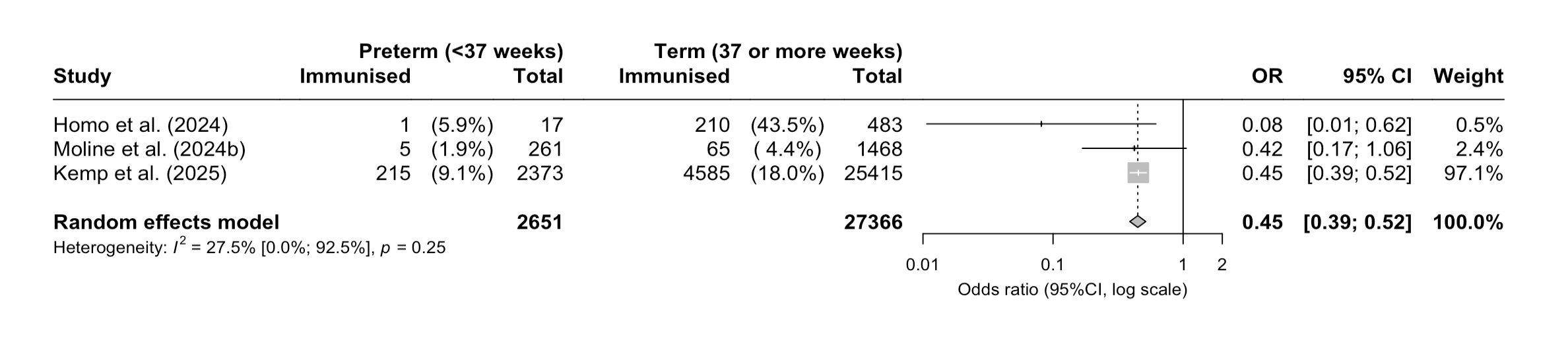


^a^ Preterm: born under 37 weeks of gestation; term: born at 37 or more weeks of gestation (ref.).

Figure S26. Uptake of RSV maternal vaccine among eligible parents of children in the United States during the 2023/24 RSV season stratified by infants’ sex assigned at birth: female (ref.) and male.


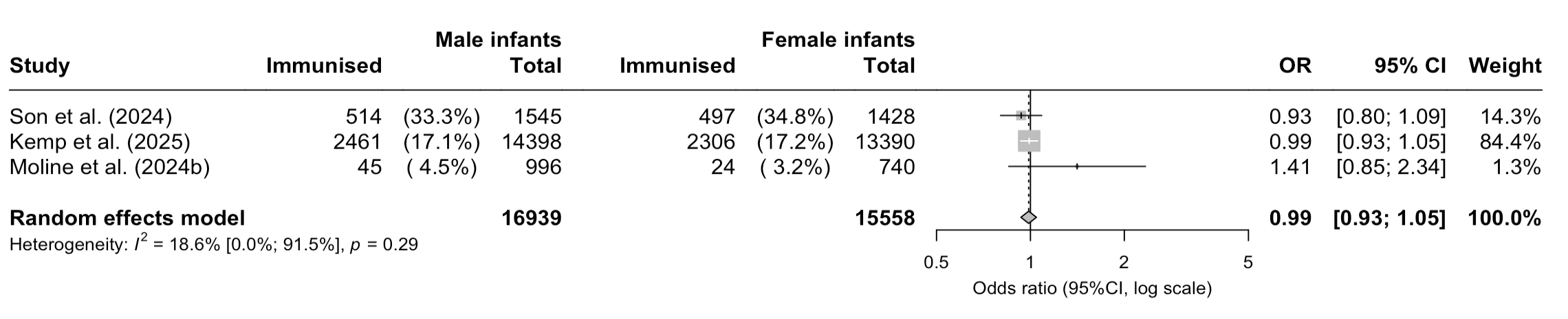


Figure S27. Uptake of RSV maternal vaccine among eligible parents of children in the United States during the 2023/24 RSV season stratified by health insurance type^a^


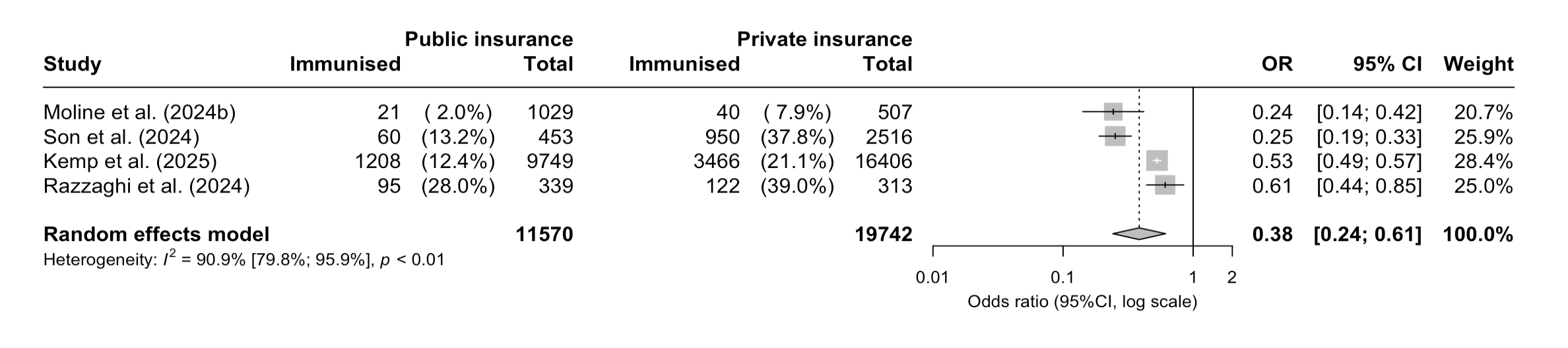
^a^ Private (or military) insurance (ref.) compared to public insurance.

Figure S28. Uptake of RSV maternal vaccine among eligible parents of children in the United States during the 2023/24 RSV season stratified by health insurance type^a^


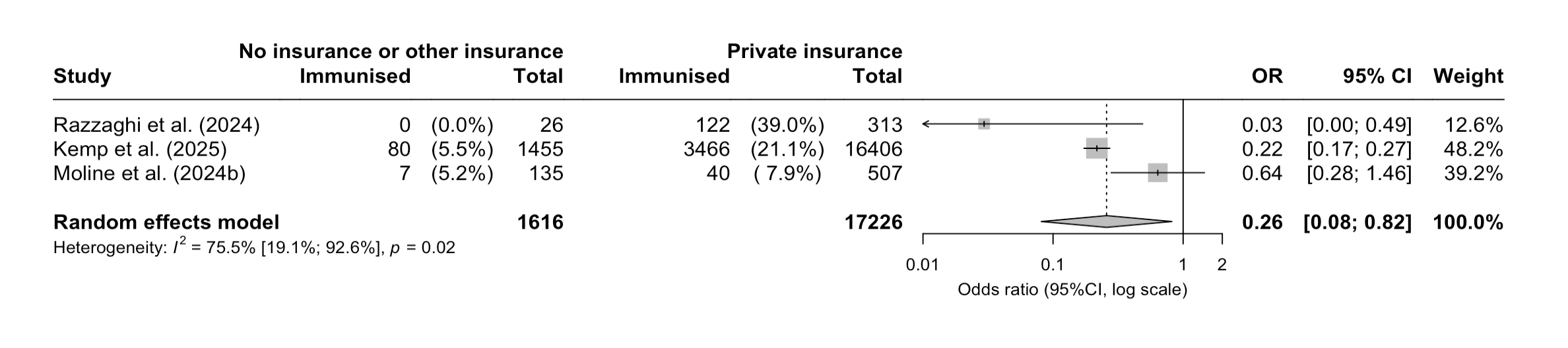


^a^ Private (or military) insurance (ref.) compared to no or other insurance.

Figure S29. Uptake of RSV maternal vaccine among eligible parents of children in the United States during the 2023/24 RSV season stratified by health insurance type^a^


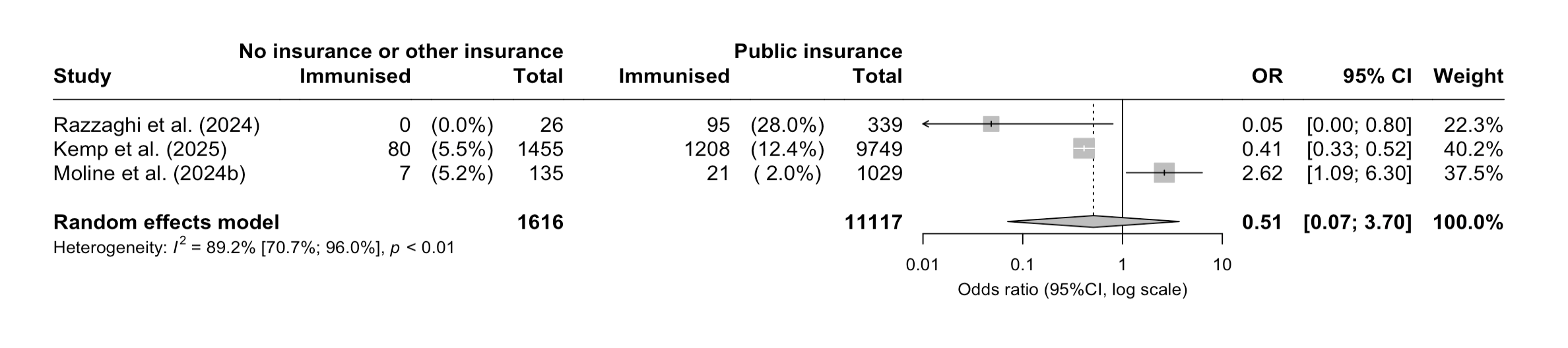


^a^ Public insurance (ref.) compared to no or other insurance.

Figure S30. Uptake of RSV maternal vaccine among eligible parents of children in the United States during the 2023/24 RSV season stratified by racial group^a^


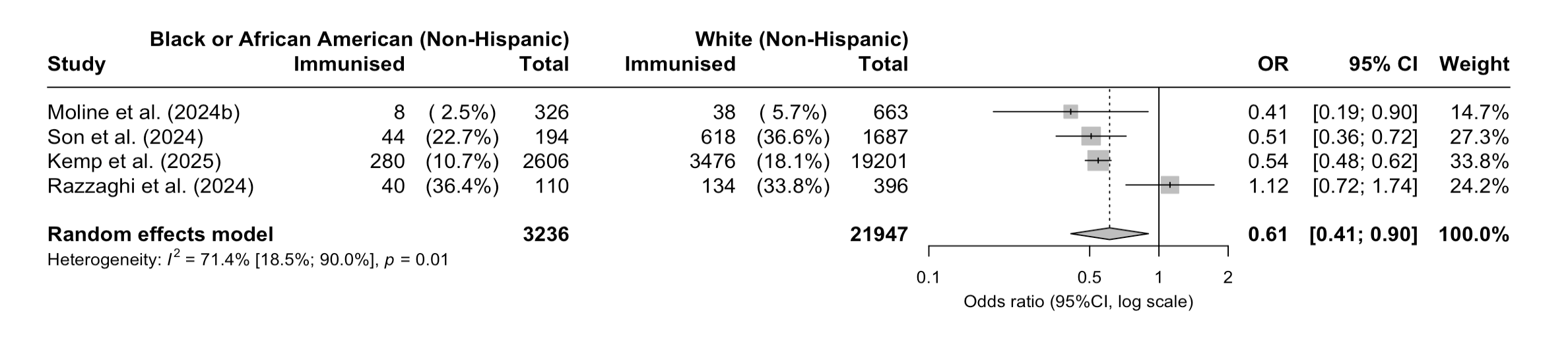


^a^ White (non-Hispanic) population (ref.) compared to Black or African American (non-Hispanic) population.

Figure S31. Uptake of RSV maternal vaccine among eligible parents of children in the United States during the 2023/24 RSV season stratified by racial group^a^


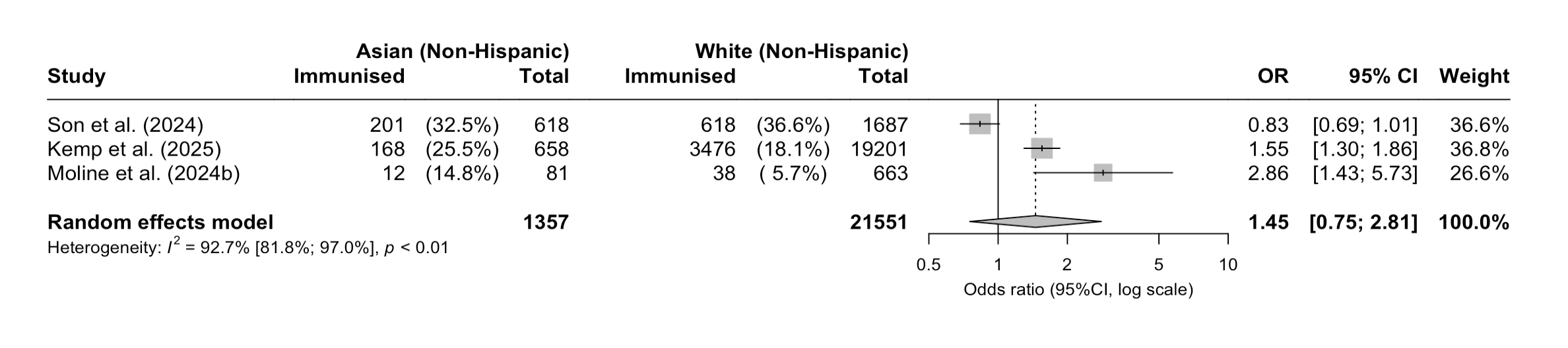


^a^ White (non-Hispanic) population (ref.) compared to Asian (non-Hispanic) population.

Figure S32. Uptake of RSV maternal vaccine among eligible parents of children in the United States during the 2023/24 RSV season stratified by racial group^a^


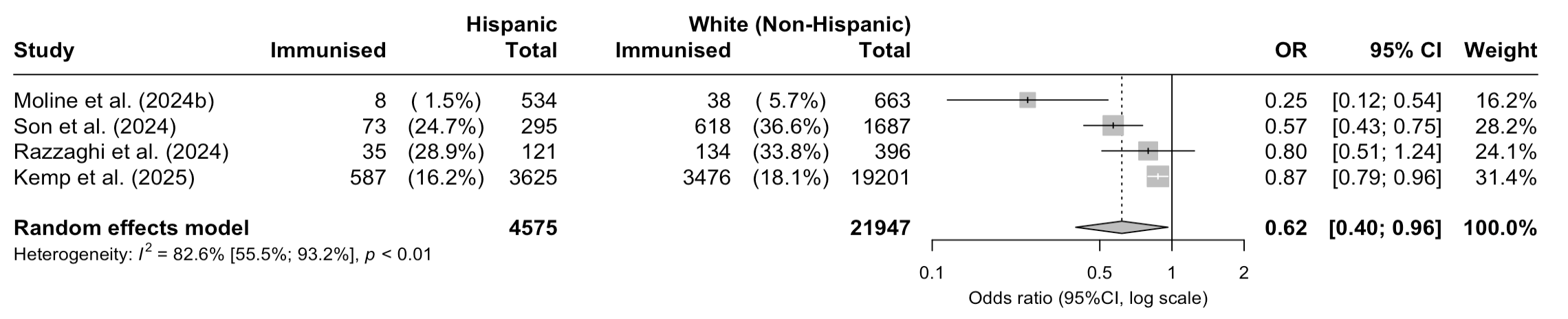


^a^ White (non-Hispanic) population (ref.) compared to Hispanic population.

Figure S33. Uptake of RSV maternal vaccine among eligible parents of children in the United States during the 2023/24 RSV season stratified by racial group^a^


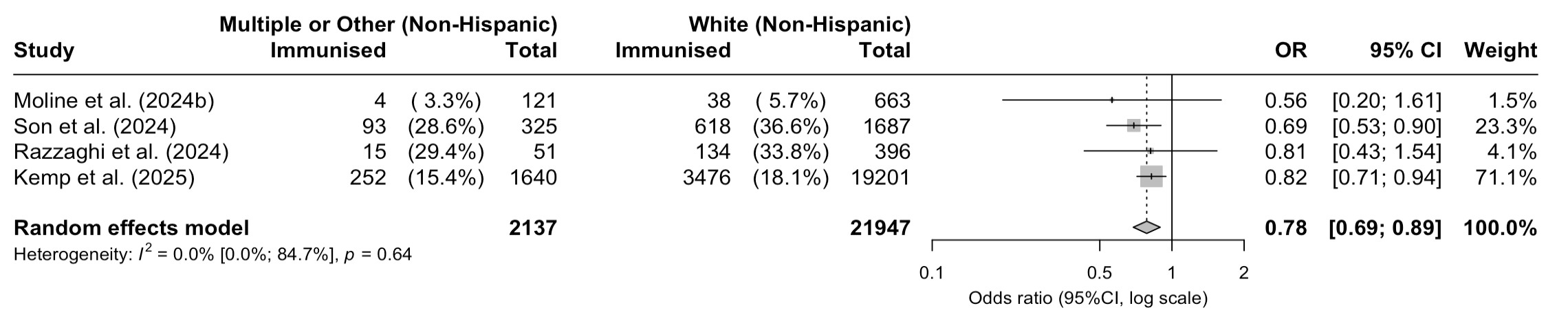
^a^ White (non-Hispanic) population (ref.) compared to population groups defined as multiple or other in the included studies.

Figure S34. Uptake of RSV maternal vaccine among eligible parents of children in the United States during the 2023/24 RSV season stratified by racial group^a^


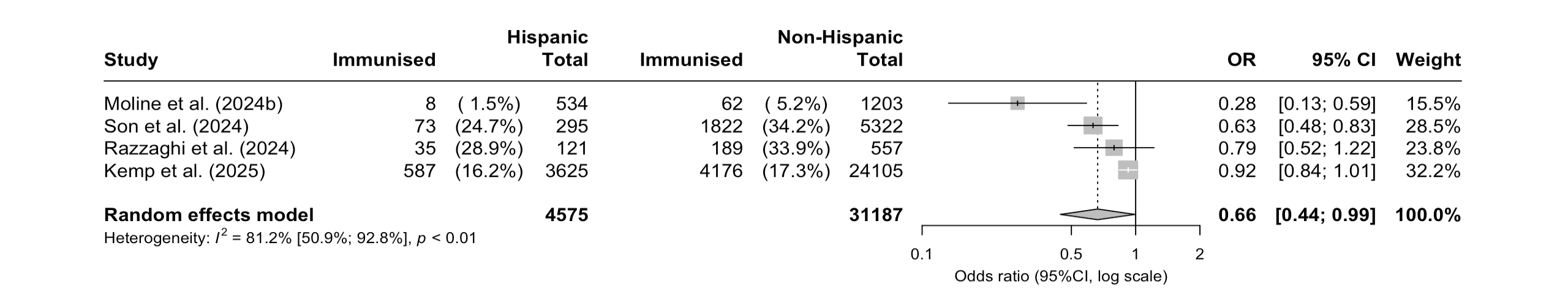


^a^ All non-Hispanic population (ref.) compared to Hispanic population.

## Uptake of RSV vaccines for older adults in the United States

Figure S35. Uptake of RSV vaccines among older adults in the United States during the 2023/24 RSV season stratified by age group^a^


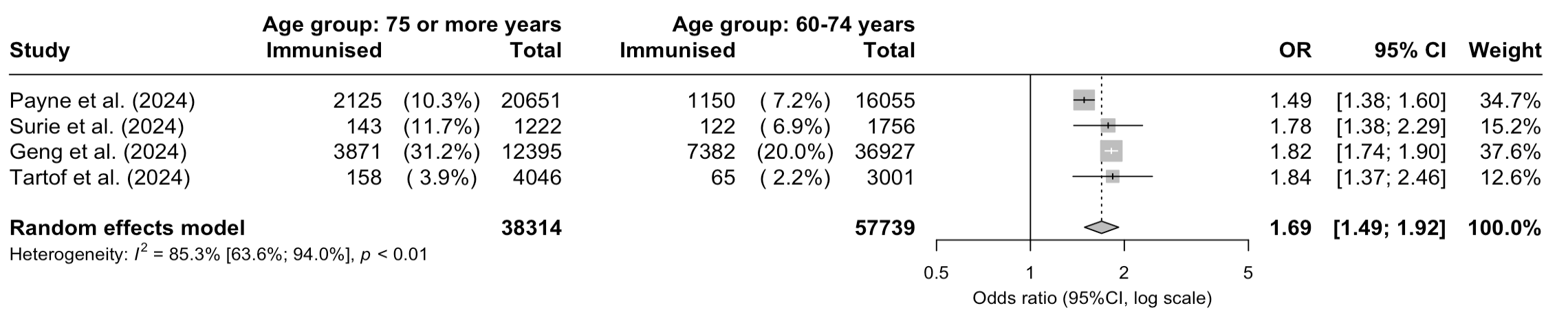
^a^ People 75 or more years old compared to people 60-74 years old (ref.).

Figure S36. Uptake of RSV vaccines among older adults in the United States during the 2023/24 RSV season stratified by sex: males and females (ref.).


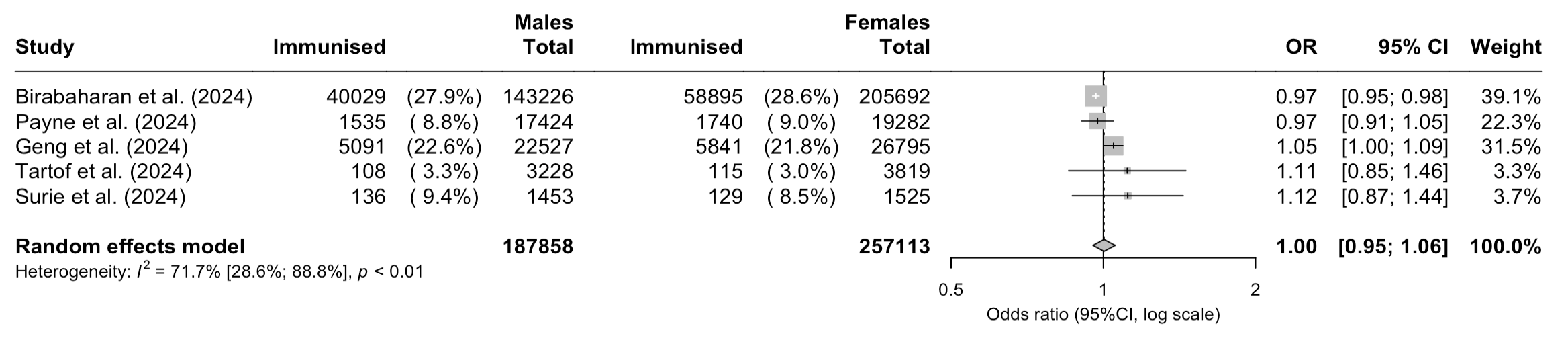


Figure S37. Uptake of RSV vaccines among older adults in the United States during the 2023/24 RSV season stratified by immunocompetence^a,b^


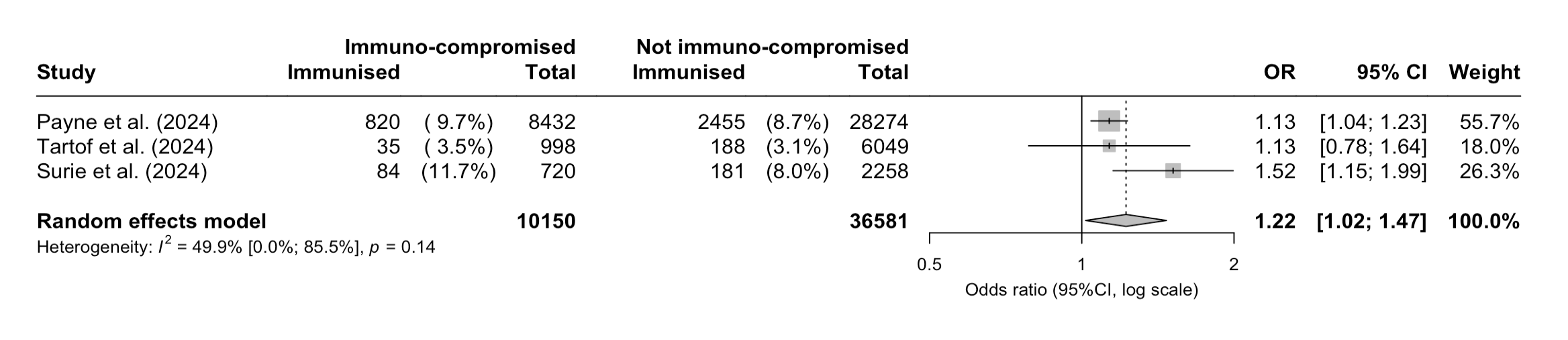
^a^ Immuno-compromised people compared to people without immune-compromised status.

^b^ In the included studies, immunocompromised status was defined as having an active solid tumour or hematologic malignancy, solid organ transplant hematopoietic cell transplant, HIV infection, primary immunodeficiency, splenectomy, use of immunosuppressive medication in the past 30 days, or other conditions that cause moderate or severe immunosuppression.

Figure S38. Uptake of RSV vaccines among eligible older adults in the United States during the 2023/24 RSV season stratified by presence of comorbidities^a,b^


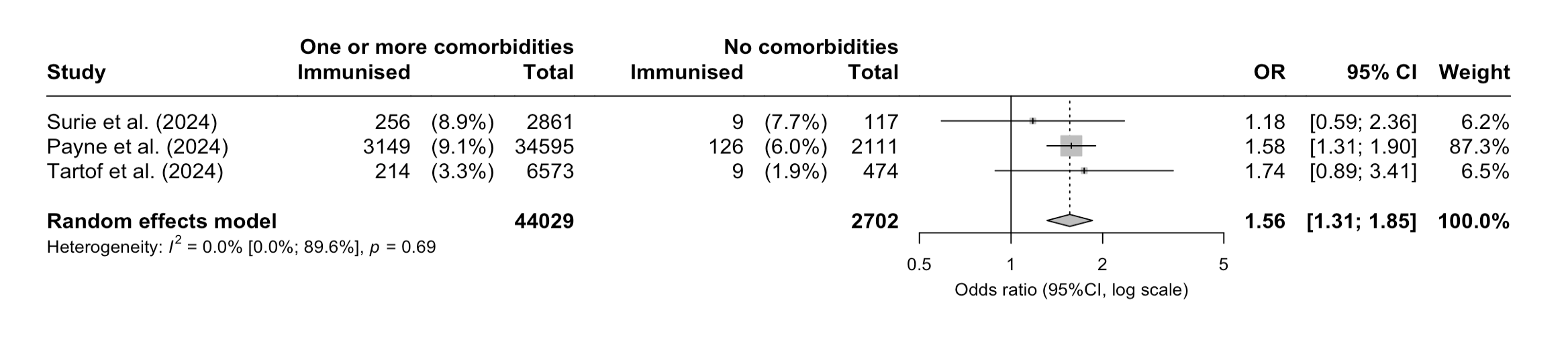


^a^ People with no comorbidities (ref.) compared to people with one or more comorbidities.

^b^ In the included studies, comorbidities were defined as cardiovascular disease (including heart failure, peripheral vascular disease that limits mobility, prior myocardial infarction, cardiac arrhythmias including atrial fibrillation and ventricular arrhythmias, valvular heart disease, hypertension, pulmonary embolism), pulmonary disease (asthma, chronic obstructive pulmonary disease, cystic fibrosis, pulmonary hypertension, home oxygen use except at night for sleep disorder, tracheostomy, home non-invasive ventilation use except at night for sleep disorder, home invasive ventilation use), neurologic disease (dementia, Down’s syndrome, prior stroke, prior transient ischemic attack, brain or spinal cord injury with loss of limb function, cerebral palsy, muscular dystrophy, multiple sclerosis, myasthenia gravis, anterolateral sclerosis), endocrine disease (diabetes mellitus with or without end organ damage, adrenal insufficiency, hypothyroidism), kidney disease (chronic kidney disease without chronic replacement therapy, end stage renal disease on chronic kidney replacement therapy), gastrointestinal disease (feeding through a tube, inflammatory bowel disease, including Crohn’s disease or ulcerative colitis, cirrhosis, chronic liver disease without cirrhosis, peptic ulcer disease), hematologic disease (sickle cell disease, coagulopathy or other bleeding disorder, chronic anaemia, thalassemia), immunocompromising conditions (active solid tumour or hematologic malignancy, solid organ transplant hematopoietic cell transplant, HIV infection, primary immunodeficiency, splenectomy, use of immunosuppressive medication in the past 30 days, or other conditions that cause moderate or severe immunosuppression), clinical obesity, clinically underweight, developmental disabilities (ADHD, autism spectrum disorders, cerebral palsy, epilepsy, intellectual disabilities and related conditions, learning disabilities, other developmental delays, spina bifida and other congenital anomalies of the nervous system), and sensory impairments (blindness and visual impairment).

Figure S39. Uptake of nirsevimab among eligible older adults in the United States during the 2023/24 RSV season stratified by racial group^a^


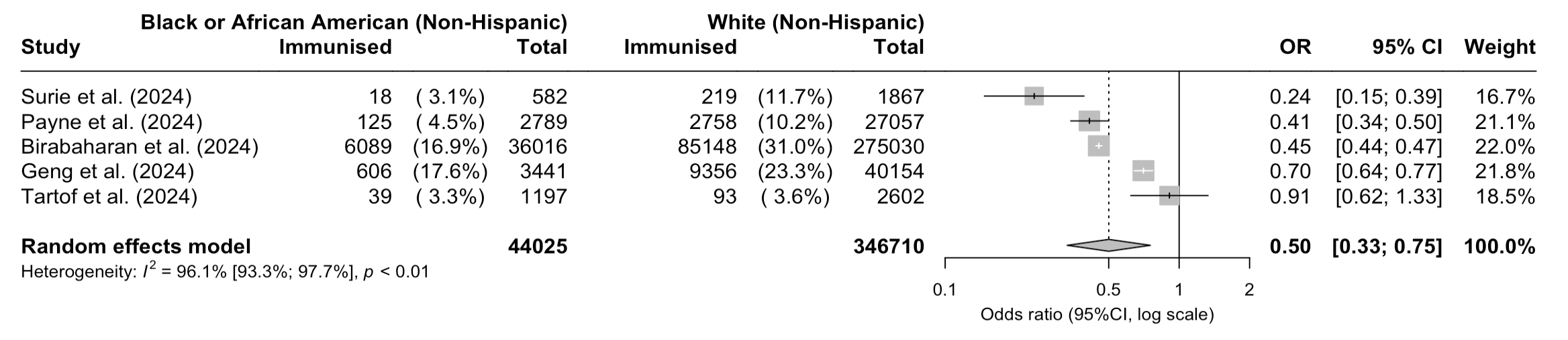
^a^ White (non-Hispanic) population (ref.) compared to Black or African American (non-Hispanic) population.

Figure S40. Uptake of nirsevimab among eligible older adults in the United States during the 2023/24 RSV season stratified by racial group^a^


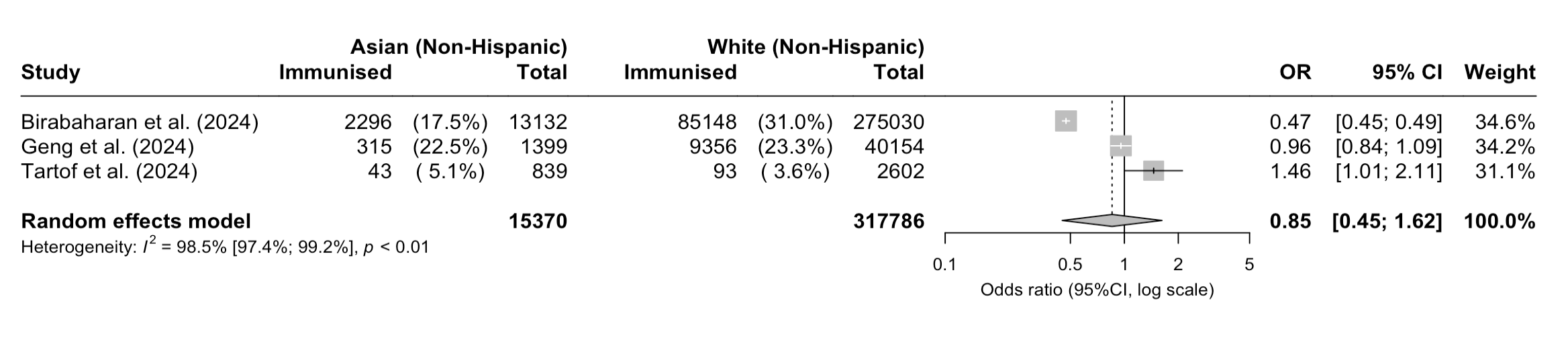


^a^ White (non-Hispanic) population (ref.) compared to Asian (non-Hispanic) population.

Figure S41. Uptake of nirsevimab among eligible older adults in the United States during the 2023/24 RSV season stratified by racial group^a^


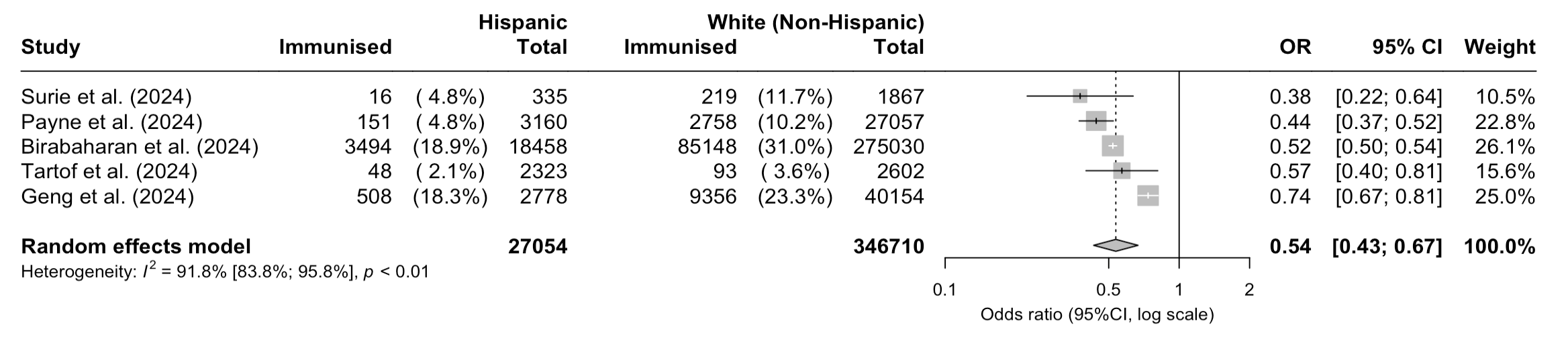
^a^ White (non-Hispanic) population (ref.) compared to Hispanic population.

Figure S42. Uptake of nirsevimab among eligible older adults in the United States during the 2023/24 RSV season stratified by racial group^a^


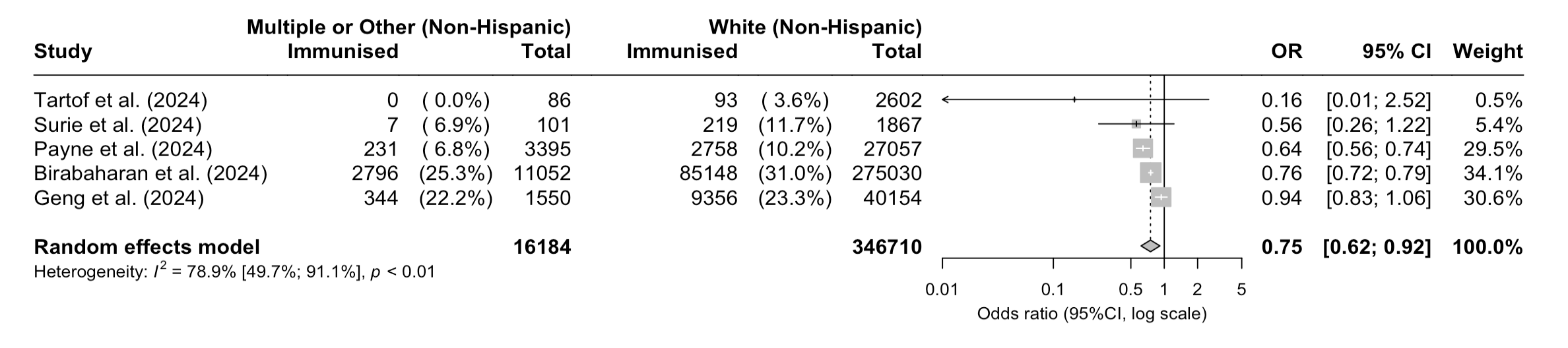
^a^ White (non-Hispanic) population (ref.) compared to population groups defined as multiple or other in the included studies.

Figure S43. Uptake of nirsevimab among eligible older adults in the United States during the 2023/24 RSV season stratified by ethnic group^a^


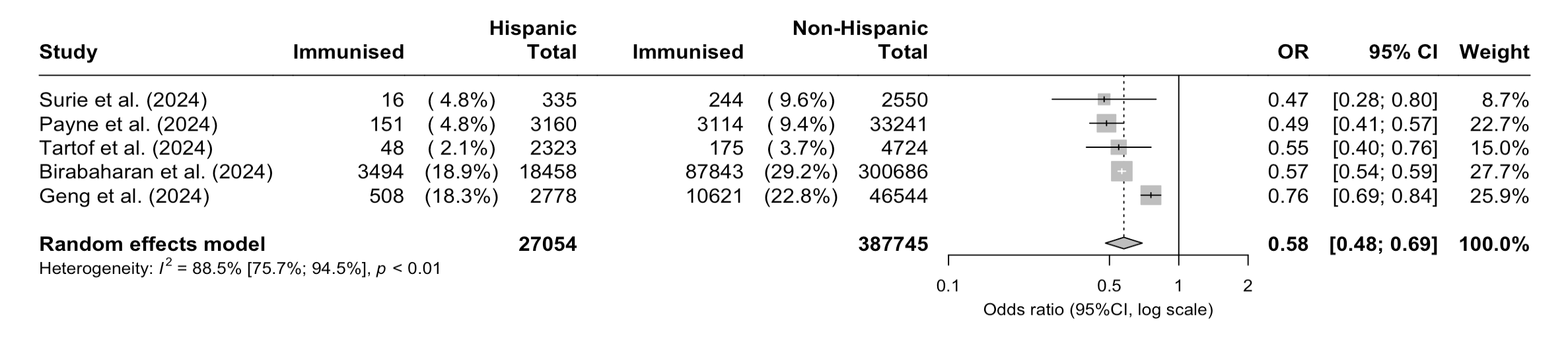


^a^ All non-Hispanic population (ref.) compared to Hispanic population.

# Supplement 7: Data used to create 'Uptake of RSV immunisation products' dashboard

Table S9: Data used to create 'Uptake of RSV immunisation products' dashboard

| **Study** | **Intervention** | **Country** | **Region** | **Total** | **Uptake** | **95% CI** | |
| --- | --- | --- | --- | --- | --- | --- | --- |
| Chauvel et al. (2024) | Nirsevimab | France | Lyon | 41 | 51.2% |  |  |
| Assad et al. (2024) | Nirsevimab | France |  | 690 | 8.7% |  |  |
| Paireau et al. (2024) | Nirsevimab | France |  | 288 | 20.1% |  |  |
| Levy et al. (2024) | Nirsevimab | France |  | 399 | 15.5% |  |  |
| Cantais et al. (2024) | Nirsevimab | France | Auvergne Rhône‐Alpes | 748 | 76.5% |  |  |
| Carbajal et al. (2024) | Nirsevimab | France | Paris | 2786 | 31.6% |  |  |
| Lenglart et al. (2025) | Nirsevimab | France |  | 383 | 20.1% |  |  |
| **Pooled uptake** | **Nirsevimab** | **France** |  |  | **28.7%** | **14.3%** | **49.2%** |
|  |  |  |  |  |  |  |  |
| Espeleta-Fox et al. (2024) | Nirsevimab | Spain | Madrid | 19 | 15.8% |  |  |
| Jimeno Ruiz et al. (2024) | Nirsevimab | Spain |  | 77 | 24.7% |  |  |
| Molina Gutiérrez et al. (2024) | Nirsevimab | Spain | Madrid | 52 | 36.5% |  |  |
| Andina Martínez et al. (2024) | Nirsevimab | Spain |  | 608 | 54.4% |  |  |
| Alejandre et al. (2024) | Nirsevimab | Spain | Catalonia | 52 | 67.3% |  |  |
| López-Lacort et al. (2024) | Nirsevimab | Spain | Valencia, Murcia and Valladolid | 166 | 69.3% |  |  |
| Barbas del Buey et al. (2024) | Nirsevimab | Spain | Madrid | 37281 | 79.6% |  |  |
| Coma et al. (2024) | Nirsevimab | Spain | Catalonia | 26525 | 87.2% |  |  |
| López-Lacort et al. (2025) | Nirsevimab | Spain | Valencia, Murcia and Valladolid | 160 | 88.1% |  |  |
| Pérez Martín and Zornoza Moreno (2024) | Nirsevimab | Spain | Valencia, Murcia and Valladolid | 13086 | 88.3% |  |  |
| Estrella-Porter et al. (2024) | Nirsevimab | Spain | Valencia, Murcia and Valladolid | 27362 | 88.5% |  |  |
| Perramon-Malavez et al. (2025) | Nirsevimab | Spain | Catalonia | 15341 | 91.6% |  |  |
| Ezpeleta et al. (2024) | Nirsevimab | Spain | Navarre | 1177 | 92.0% |  |  |
| Mallah et al. (2024) | Nirsevimab | Spain | Galicia | 14476 | 92.0% |  |  |
| Ares-Gómez et al. (2024) | Nirsevimab | Spain | Galicia | 360 | 96.7% |  |  |
| **Pooled uptake** | **Nirsevimab** | **Spain** |  |  | **78.0%** | **63.5%** | **87.8%** |
|  |  |  |  |  |  |  |  |
| Homo et al. (2024) | Nirsevimab | USA | South | 289 | 76.1% |  |  |
| Kemp et al. (2025) | Nirsevimab | USA | Northeast | 27788 | 18.2% |  |  |
| Lefferts et al. (2024) | Nirsevimab | USA | West | 1591 | 47.5% |  |  |
| Moline et al. (2024a) | Nirsevimab | USA | Northeast | 235 | 3.8% |  |  |
| Moline et al. (2024a) | Nirsevimab | USA | West | 176 | 9.7% |  |  |
| Moline et al. (2024a) | Nirsevimab | USA | South | 288 | 11.5% |  |  |
| Moline et al. (2024b) | Nirsevimab | USA | Northeast | 512 | 4.7% |  |  |
| Moline et al. (2024b) | Nirsevimab | USA | Midwest | 512 | 4.7% |  |  |
| Moline et al. (2024b) | Nirsevimab | USA | Midwest | 402 | 6.7% |  |  |
| Moline et al. (2024b) | Nirsevimab | USA | West | 207 | 9.7% |  |  |
| Moline et al. (2024b) | Nirsevimab | USA | West | 207 | 9.7% |  |  |
| Moline et al. (2024b) | Nirsevimab | USA | South | 495 | 13.1% |  |  |
| Moline et al. (2024b) | Nirsevimab | USA | South | 495 | 13.1% |  |  |
| Puckett et al. (2025) | Nirsevimab | USA | West | 1110 | 71.5% |  |  |
| Razzaghi et al. (2024) | Nirsevimab | USA | West | 159 | 36.5% |  |  |
| Razzaghi et al. (2024) | Nirsevimab | USA | Northeast | 116 | 44.0% |  |  |
| Razzaghi et al. (2024) | Nirsevimab | USA | South | 379 | 47.5% |  |  |
| Razzaghi et al. (2024) | Nirsevimab | USA | Midwest | 212 | 48.6% |  |  |
| Xu et al. (2024) | Nirsevimab | USA | Northeast | 3090 | 10.7% |  |  |
| **Pooled uptake** | **Nirsevimab** | **USA** |  |  | **30.4%** | **13.7%** | **54.5%** |
|  |  |  |  |  |  |  |  |
| **Agüera et al. (2024)** | **Nirsevimab** | **Spain and Andorra** |  | **181** | **60.2%** |  |  |
|  |  |  |  |  |  |  |  |
| **Ernst et al. (2024)** | **Nirsevimab** | **Luxembourg** | **Luxembourg** | **1524** | **83.8%** |  |  |
|  |  |  |  |  |  |  |  |
| **Consolati et al. (2024)** | **Nirsevimab** | **Italy** | **Valle d' Aosta** | **537** | **68.70%** |  |  |
|  |  |  |  |  |  |  |  |
| Kemp et al. (2025) | Maternal vaccine | USA | Northeast | 27788 | 17.2% |  |  |
| Moline et al. (2024b) | Maternal vaccine | USA |  | 1737 | 4.0% |  |  |
| Son et al. (2024) | Maternal vaccine | USA | Northeast | 2973 | 34.0% |  |  |
| Homo et al. (2024) | Maternal vaccine | USA | South | 500 | 42.2% |  |  |
| Razzaghi et al. (2024) | Maternal vaccine | USA | Northeast | 86 | 38.4% |  |  |
| Razzaghi et al. (2024) | Maternal vaccine | USA | Midwest | 163 | 36.8% |  |  |
| Razzaghi et al. (2024) | Maternal vaccine | USA | South | 299 | 29.8% |  |  |
| Razzaghi et al. (2024) | Maternal vaccine | USA | West | 130 | 30.0% |  |  |
| **Pooled uptake** | **Maternal vaccine** | **USA** |  |  | **21.6%** | **9.2%** | **42.9%** |
|  |  |  |  |  |  |  |  |
| Tartof et al. (2024) | Vaccine for older adults | USA | West | 7047 | 3.2% |  |  |
| Surie et al. (2024) | Vaccine for older adults | USA |  | 2978 | 8.9% |  |  |
| Payne et al. (2024) | Vaccine for older adults | USA |  | 36706 | 8.9% |  |  |
| Reses et al. (2023) | Vaccine for older adults | USA |  | 238449 | 9.8% |  |  |
| Motta et al. (2025) | Vaccine for older adults | USA |  | 358 | 14.0% |  |  |
| Geng et al. (2024) | Vaccine for older adults | USA | South | 16114 | 20.0% |  |  |
| Geng et al. (2024) | Vaccine for older adults | USA | Northeast | 7366 | 21.7% |  |  |
| Geng et al. (2024) | Vaccine for older adults | USA | Midwest | 10214 | 23.0% |  |  |
| Geng et al. (2024) | Vaccine for older adults | USA | West | 15628 | 25.4% |  |  |
| Birabaharan et al. (2024) | Vaccine for older adults | USA |  | 357814 | 27.9% |  |  |
| **Pooled uptake** | **Vaccine for older adults** | **USA** |  |  | **11.8%** | **6.6%** | **20.0%** |

# References

1. Blauvelt CA, Zeme M, Natarajan A, et al. Respiratory Syncytial Virus Vaccine and Nirsevimab Uptake Among Pregnant People and Their Neonates. *JAMA Network Open* 2025; **8**(2): e2460735-e.

2. Georgiadis T, Poupouzas GI, Athanasopoulou G, et al. Characteristics and outcomes of RSV and Influenza hospitalized patients. *European Respiratory Journal* 2024; **64**(suppl 68): PA5112.

3. Hamid O, Mohammed SS, Awadalla M, Hammad F, Regueiro MD. Respiratory Syncytial Virus Vaccine is Associated With Better Outcomes in Inflammatory Bowel Disease Patients over 60 Years Old: A US Propensity-Matched Study. *Official journal of the American College of Gastroenterology | ACG* 2024; **119**(10S).

4. Hsiao A, Hansen J, Timbol J, et al. Effectiveness of nirsevimab in infants against respiratory syncytial virus and related events. *Annals of Allergy, Asthma & Immunology* 2024; **133**(6, Supplement 2): S3-S4.

5. La E, McGuiness C, Singer D, Yasuda M, Chen C. Characteristics associated with respiratory syncytial virus vaccination among adults aged 60 years and older in the United States. *JMCP* 2024; **30**(10-c Suppl): S1-S165.

6. Lai X, Ma Y, Zou W, Soudani S, Fang H. Public Health Impact of Nirsevimab Against Lower Respiratory Infections Associated with Respiratory Syncytial Virus Among Chinese Infants. *Value in Health* 2023; **26**(12): S241.

7. Loeb L, Henkes N, Picco M, Kinnucan J, Hashash J, Farraye F. Outcomes of respiratory syncytial virus infections in patients with inflammatory bowel disease. *Gastroenterology* 2024; **166**: S-1449.

8. Lorenzini G, Curran D, Matthews S, et al. Respiratory Syncytial Virus Candidate Vaccine Attenuates the Severity of Breakthrough Infections. *Respiration* 2023; **102**(8): 636-802.

9. Martin M, Woods J, Austin V, Shone J, Connell D. The incidence and impact of influenza, RSV and SARS-CoV2 on a Scottish Health Board between 2022 and 2024. *Thorax* 2024; **79**(Suppl 2): A112.

10. Molnar D, La EM, Verelst F, et al. Assessing the Public Health Impact of the Adjuvanted Respiratory Syncytial Virus Prefusion F Protein Vaccine Among Older Adults in the United States (US). *Value in Health* 2023; **26**(6, Supplement): S172.

11. Rallabhandi SSH, Salman A, Schultz B, Thameem D. Understanding the current prevalence of RSV: Exploring associations with immunization, infection and hospitalization trends. *Chest* 2024; **166**(4): A3923.

12. Adhikari S, Chapagain RH, Maharjan J, et al. Acceptance of New Respiratory Syncytial Virus Vaccine among Pregnant Women in Nepal for Future Routine Immunization: A Descriptive Crosssectional Study. *Journal of Nepal Medical Association* 2024; **62**: 372-7.

13. Brault A, Pontais I, Enouf V, et al. Effect of nirsevimab on hospitalisations for respiratory syncytial virus bronchiolitis in France, 2023-24: a modelling study. *The Lancet Child & adolescent health* 2024; **8**(10): 721-9.

14. Du Z, Pandey A, Moghadas SM, et al. Impact of RSVpreF vaccination on reducing the burden of respiratory syncytial virus in infants and older adults. *Nature Medicine* 2025; **31**(2): 647-52.

15. Hansen CL, Lee L, Bents SJ, et al. Scenario projections of RSV hospitalizations averted due to new immunization programs in King County, Washington, October 2023 to May 2025. *medRxiv* 2024: 2024.12.13.24319008.

16. Maculaitis MC, Hauber B, Beusterien KM, et al. A latent class analysis of factors influencing preferences for infant respiratory syncytial virus (RSV) preventives among pregnant people in the United States. *Human vaccines & immunotherapeutics* 2024; **20**(1): 2358566.

17. Mazagatos C, Mendioroz J, Rumayor MB, et al. Estimated Impact of Nirsevimab on the Incidence of Respiratory Syncytial Virus Infections Requiring Hospital Admission in Children < 1 Year, Weeks 40, 2023, to 8, 2024, Spain. *Influenza and other Respiratory Viruses* 2024; **18**(5): e13294.

18. Sallam M, Kherfan T, Al-Farajat A, et al. Attitude to RSV Vaccination Among a Cohort of Pregnant Women in Jordan: A Cross-Sectional Survey Study. *Health Science Reports* 2025; **8**(1): e70319.

19. Trubin P, Azar MM, Kotton CN. The respiratory syncytial virus vaccines are here: Implications for solid organ transplantation. *American Journal of Transplantation* 2024; **24**(6): 897-904.

20. Alami A, Perez-Lloret S, Mattison DR. Safety of RSV Vaccine among Pregnant Individuals: A Real-World Pharmacovigilance Study Using Vaccine Adverse Event Reporting System. *medRxiv* 2024; ((Alami, Mattison) School of Epidemiology and Public Health, Faculty of Medicine, University of Ottawa, Ottawa, ON, Canada(Perez-Lloret) Consejo Nacional de Investigaciones Cientificas y Tecnicas (CONICET), Buenos Aires, Argentina(Perez-Lloret) Observatori).

21. Carcione D, Spencer P, Pettigrew G, et al. Active post-marketing safety surveillance of nirsevimab administered children in Western Australia, April-July 2024. *The Pediatric Infectious Disease Journal* 9900.

22. Domnich A, Orsi A, Lai PL, et al. Characteristics of the First Italian Older Adults Vaccinated with an Adjuvanted Respiratory Syncytial Virus (RSV) Vaccine. *Medicina* 2025; **61**(1).

23. Falsey AR, Branche AR, Peasley M, et al. Short-Term Immunogenicity of Licensed Subunit RSV Vaccines in Residents of Long-Term Care Facilities (LTCF) Compared to Community-Dwelling Older Adults. *Journal of the American Medical Directors Association* 2024; **25**(11): 105281.

24. Perramon-Malavez A, de Rioja VL, Coma E, et al. Introduction of nirsevimab in Catalonia, Spain: description of the incidence of bronchiolitis and respiratory syncytial virus in the 2023/2024 season. *European Journal of Pediatrics* 2024; ((Perramon-Malavez, de Rioja, Prats) Computational Biology and Complex Systems (BIOCOM-SC) Group, Department of Physics, Universitat Politecnica de Catalunya (UPC), Catalonia, Castelldefels, Barcelona, Spain(Coma, Hermosilla, Fina) Primary Care Services In).

25. Mestre-Ferrándiz J, Rivero A, Orrico-Sánchez A, et al. Evaluation of antibody-based preventive alternatives for respiratory syncytial virus: a novel multi-criteria decision analysis framework and assessment of nirsevimab in Spain. *BMC Infectious Diseases* 2024; **24**(1): 99.

26. Bracaloni S, Esposito E, Scarpaci M, et al. RSV Disease Burden in Older Adults: An Italian Multiregion Pilot Study of Acute Respiratory Infections in Primary Care Setting, Winter Season 2022–2023. *Influenza and other Respiratory Viruses* 2024; **18**.

27. Grahić-Mujčinović O, Smajlović E, Tabaković S, Alić A. Palivizumab in the prophylaxis of respiratory syncytial virus infections. *BMJ Paediatrics Open* 2024; **8**(Suppl 5): null.

28. Moro PL, Gallego R, Scheffey A, et al. Administration of the GSK Respiratory Syncytial Virus Vaccine to Pregnant Persons in Error. *Obstetrics and gynecology* 2024; **143**(5): 704-6.

29. Raguž MJ, Božić T, Nikše T. Is immunization with palivizumab really effective in high-risk children? *J Mother Child* 2022; **26**(1): 87-92.

30. Remmele J, Helm PC, Li J, Oberhoffer-Fritz R, Bauer UMM, Ewert P. Twins with at least one with CHD and their immunisation status in direct comparison—are both twins complying with the German immunisation recommendations? *Cardiovascular Diagnosis and Therapy* 2024; **14**(6): 1108121-1121.

31. Levy C, Werner A, Rybak A, et al. Early Impact of Nirsevimab on Ambulatory All-Cause Bronchiolitis: A Prospective Multicentric Surveillance Study in France. *Journal of the Pediatric Infectious Diseases Society* 2024; **13**(7): 371-3.

32. López-Lacort M, Muñoz-Quiles C, Mira-Iglesias A, et al. Nirsevimab Effectiveness Against Severe Respiratory Syncytial Virus Infection in the Primary Care Setting. *Pediatrics* 2025; **155**(1): e2024066393.

33. Moline HL, Toepfer AP, Tannis A, et al. Respiratory Syncytial Virus Disease Burden and Nirsevimab Effectiveness in Young Children From 2023-2024. *JAMA Pediatrics* 2025; **179**(2): 179-87.

34. Rodríguez-Fernández R, González-Martínez F, Ojeda Velázquez I, et al. Nirsevimab effectiveness against hospital admission for respiratory syncytial virus bronchiolitis in infants. *Rev Esp Quimioter* 2024; **37**(6): 498-503.

35. Biegus J, Szenborn L, Zymliński R, et al. The early safety profile of simultaneous vaccination against influenza and Respiratory Syncytial Virus (RSV) in patients with high-risk heart failure. *Vaccine* 2024; **42**(12): 2937-40.

36. Domachowske J, Madhi SA, Simões EAF, et al. Safety of Nirsevimab for RSV in Infants with Heart or Lung Disease or Prematurity. *The New England journal of medicine* 2022; **386**(9): 892-4.

37. Domachowske JB, Chang Y, Atanasova V, et al. Safety of Re-dosing Nirsevimab Prior to RSV Season 2 in Children With Heart or Lung Disease. *Journal of the Pediatric Infectious Diseases Society* 2023; **12**(8): 477-80.

38. Novoa Pizarro JM, Lindemann Tappert BC, Luchsinger Farías VR, Vargas Munita SL. Prevención de la infección por virus respiratorio sincicial en lactantes. ¿Qué se ha hecho y en qué estamos hoy? *Andes pediatrica* 2023; **94**: 672-80.

39. Institute JB. Critical Appraisal Tools for Use in JBI Systematic Reviews. 2020. <https://jbi.global/critical-appraisal-tools> (accessed 24 Feb 2025).

40. Ares-Gómez S, Mallah N, Santiago-Pérez M-I, et al. Effectiveness and impact of universal prophylaxis with nirsevimab in infants against hospitalisation for respiratory syncytial virus in Galicia, Spain: initial results of a population-based longitudinal study. *The Lancet Infectious Diseases* 2024; **24**(8): 817-28.

41. Barbas Del Buey JF, Inigo Martinez J, Gutierrez Rodriguez MA, et al. The effectiveness of nirsevimab in reducing the burden of disease due to respiratory syncytial virus (RSV) infection over time in the Madrid region (Spain): a prospective population-based cohort study. *Frontiers in public health* 2024; **12**((Barbas Del Buey) FIIBAP Fundacion para la Investigacion e Innovacion Biosanitaria de Atencion Primaria, Madrid, Spain(Barbas Del Buey, Inigo Martinez, Gutierrez Rodriguez, Alonso Garcia, Sanchez-Gomez, Lasheras Carbajo, Jimenez Bueno, Esteban Vasallo, Lo): 1441786.

42. Birabaharan M, Johns ST, Kaelber DC, Martin TCS, Mehta SR. Atrial Fibrillation after RSV Vaccination Among Older Adults. *Clin Infect Dis* 2024.

43. Coma E, Martinez-Marcos M, Hermosilla E, et al. Effectiveness of nirsevimab immunoprophylaxis against respiratory syncytial virus-related outcomes in hospital and primary care settings: a retrospective cohort study in infants in Catalonia (Spain). *Archives of disease in childhood* 2024; **109**(9): 736-41.

44. Estrella-Porter P, Blanco-Calvo C, Lameiras-Azevedo AS, et al. Effectiveness of nirsevimab introduction against respiratory syncytial virus in the Valencian Community: A preliminary assessment. *Vaccine* 2024; **42**(22): 126030.

45. Ezpeleta G, Navascués A, Viguria N, et al. Effectiveness of Nirsevimab Immunoprophylaxis Administered at Birth to Prevent Infant Hospitalisation for Respiratory Syncytial Virus Infection: A Population-Based Cohort Study. *Vaccines* 2024; **12**(4): 383.

46. Homo RL, Groberg A, Donahue M, Halverson D, Wooten A, Ponnapakkam A. High Uptake of Respiratory Syncitial Virus Prevention for Neonates in a Military Treatment Facility. *The Journal of Pediatrics* 2024; **273**.

47. Jimeno Ruiz S, Peláez A, Labourt A, et al. Evaluating the Effectiveness of Nirsevimab in Reducing Pediatric RSV Hospitalizations in Spain. *Vaccines* 2024; **12**(10): 1160.

48. Mallah N, Pardo-Seco J, Pérez-Martínez O, et al. Full 2023–24 season results of universal prophylaxis with nirsevimab in Galicia, Spain: the NIRSE-GAL study. *The Lancet Infectious Diseases* 2024; **0**(0).

49. Martinón-Torres F, Mirás-Carballal S, Durán-Parrondo C. Early lessons from the implementation of universal respiratory syncytial virus prophylaxis in infants with long-acting monoclonal antibodies, Galicia, Spain, September and October 2023. *Eurosurveillance* 2023; **28**(49): 2300606.

50. Puckett L, Kushner LE, Bio L, Cornell S, Wood M, Schwenk HT. Successful Implementation of Nirsevimab and Factors Influencing Uptake in Neonatal Care. *Hospital Pediatrics* 2025; **15**(2): 99-107.

51. Reses HE. Coverage with Influenza, Respiratory Syncytial Virus, and Updated COVID-19 Vaccines Among Nursing Home Residents — National Healthcare Safety Network, United States, December 2023. *MMWR Morbidity and mortality weekly report* 2023; **72**.

52. Reses HE. Coverage with Influenza, Respiratory Syncytial Virus, and COVID-19 Vaccines Among Nursing Home Residents — National Healthcare Safety Network, United States, November 2024. *MMWR Morbidity and mortality weekly report* 2024; **73**.

53. Son M, Riley LE, Staniczenko AP, et al. Nonadjuvanted Bivalent Respiratory Syncytial Virus Vaccination and Perinatal Outcomes. *JAMA Network Open* 2024; **7**(7): e2419268.

54. Geng X, Wang W. Respiratory syncytial virus vaccination among US adults aged ≥60 years. *Front Immunol* 2024; **15**: 1427550.

55. Kemp M, Capriola A, Schauer S. RSV immunization uptake among infants and pregnant persons — Wisconsin, October 1, 2023-March 31, 2024. *Vaccine* 2025; **47**: 126674.

56. Motta M, Callaghan T, Padmanabhan M, et al. Quantifying the prevalence and determinants of respiratory syncytial virus (RSV) vaccine hesitancy in US adults aged 60 or older. *Public Health* 2025; **238**: 3-6.

57. Pérez Martín JJ, Zornoza Moreno M. Implementation of the first respiratory syncytial (RSV) immunization campaign with nirsevimab in an autonomous community in Spain. *Human Vaccines & Immunotherapeutics* 2024; **20**(1): 2365804.

58. Razzaghi H. Maternal Respiratory Syncytial Virus Vaccination and Receipt of Respiratory Syncytial Virus Antibody (Nirsevimab) by Infants Aged 8 Months — United States, April 2024. *MMWR Morbidity and mortality weekly report* 2024; **73**.

59. Agüera M, Soler-Garcia A, Alejandre C, et al. Nirsevimab immunization's real-world effectiveness in preventing severe bronchiolitis: A test-negative case–control study. *Pediatric Allergy and Immunology* 2024; **35**(6): e14175.

60. Assad Z, Romain A-S, Aupiais C, et al. Nirsevimab and Hospitalization for RSV Bronchiolitis. *The New England journal of medicine* 2024; **391**(2): 144-54.

61. Carbajal R, Boelle P-Y, Pham A, et al. Real-world effectiveness of nirsevimab immunisation against bronchiolitis in infants: a case-control study in Paris, France. *The Lancet Child & adolescent health* 2024; **8**(10): 730-9.

62. Lefferts B. Nirsevimab Effectiveness Against Medically Attended Respiratory Syncytial Virus Illness and Hospitalization Among Alaska Native Children — Yukon-Kuskokwim Delta Region, Alaska, October 2023–June 2024. *MMWR Morbidity and mortality weekly report* 2024; **73**.

63. Lenglart L, Levy C, Basmaci R, et al. Nirsevimab effectiveness on paediatric emergency visits for RSV bronchiolitis: a test-negative design study. *European Journal of Pediatrics* 2025; **184**(2): 171.

64. López-Lacort M, Muñoz-Quiles C, Mira-Iglesias A, et al. Early estimates of nirsevimab immunoprophylaxis effectiveness against hospital admission for respiratory syncytial virus lower respiratory tract infections in infants, Spain, October 2023 to January 2024. *Eurosurveillance* 2024; **29**(6): 2400046.

65. Moline HL. Early Estimate of Nirsevimab Effectiveness for Prevention of Respiratory Syncytial Virus–Associated Hospitalization Among Infants Entering Their First Respiratory Syncytial Virus Season — New Vaccine Surveillance Network, October 2023–February 2024. *MMWR Morbidity and mortality weekly report* 2024; **73**.

66. Paireau J, Durand C, Raimbault S, et al. Nirsevimab Effectiveness Against Cases of Respiratory Syncytial Virus Bronchiolitis Hospitalised in Paediatric Intensive Care Units in France, September 2023-January 2024. *Influenza and other Respiratory Viruses* 2024; **18**(6): e13311.

67. Payne AB, Watts JA, Mitchell PK, et al. Respiratory syncytial virus (RSV) vaccine effectiveness against RSV-associated hospitalisations and emergency department encounters among adults aged 60 years and older in the USA, October, 2023, to March, 2024: a test-negative design analysis. *Lancet (London, England)* 2024; **404**(10462): 1547-59.

68. Surie D, Self WH, Zhu Y, et al. RSV Vaccine Effectiveness Against Hospitalization among US Adults 60 Years and Older. *JAMA* 2024; ((Surie, Yuengling, Dawood) Coronavirus and Other Respiratory Viruses Division, Centers for Disease Control and Prevention, Atlanta, GA, United States(Self) Vanderbilt Institute for Clinical and Translational Research, Vanderbilt University Medical Center).

69. Tartof SY, Aliabadi N, Goodwin G, et al. Estimated Vaccine Effectiveness for Respiratory Syncytial Virus–Related Lower Respiratory Tract Disease. *JAMA Network Open* 2024; **7**(12): e2450832.

70. Xu H, Aparicio C, Wats A, et al. Real-World Effectiveness of Nirsevimab Against Respiratory Syncytial Virus: A Test-Negative Case-Control Study. medRxiv; 2024.

71. Alejandre C, Penela-Sánchez D, Alsina J, et al. Impact of universal immunization program with monoclonal antibody nirsevimab on reducing the burden of serious bronchiolitis that need pediatric intensive care. *European Journal of Pediatrics* 2024; **183**(9): 3897-904.

72. Andina Martinez D, Claret Teruel G, Gijon Mediavilla M, et al. Nirsevimab and Acute Bronchiolitis Episodes in Pediatric Emergency Departments. *Pediatrics* 2024; **154**(4).

73. Cantais A, Annino N, Thuiller C, et al. First RSV epidemic with nirsevimab. Older children than previous epidemics, even when hospitalized. *Journal of Medical Virology* 2024; **96**(2): e29483.

74. Chauvel C, Horvat C, Javouhey E, et al. Changes in Respiratory Syncytial Virus-Associated Hospitalisations Epidemiology After Nirsevimab Introduction in Lyon, France. *Influenza and other Respiratory Viruses* 2024; **18**(12): e70054.

75. Consolati A, Farinelli M, Serravalle P, et al. Safety and Efficacy of Nirsevimab in a Universal Prevention Program of Respiratory Syncytial Virus Bronchiolitis in Newborns and Infants in the First Year of Life in the Valle d’Aosta Region, Italy, in the 2023–2024 Epidemic Season. *Vaccines* 2024; **12**(5): 549.

76. Ernst C, Bejko D, Gaasch L, et al. Impact of nirsevimab prophylaxis on paediatric respiratory syncytial virus (RSV)-related hospitalisations during the initial 2023/24 season in Luxembourg. *Eurosurveillance* 2024; **29**(4): 2400033.

77. Espeleta-Fox A, Garcia-Salido A, Vallespin-Casas A, et al. Impact of nirsevimab on admission to a Spanish pediatric intensive care unit because of RSV bronchiolitis: Unicentric observational study from 2017 to 2024. *Pediatric Pulmonology* 2024; ((Espeleta-Fox, Vallespin-Casas, Leoz-Gordillo, Unzueta-Roch, De Lama Caro-Paton, Garcia-Teresa, Martinez de Azagra-Garde) Pediatric Critical Care Unit, Hospital Infantil Universitario Nino Jesus, Madrid, Spain(Garcia-Salido) Pediatric Intensive Care Unit).

78. Molina Gutiérrez MÁ, de Miguel Lavisier B, Ruiz Domínguez JA, et al. Impact of nirsevimab immunization on RSV infections attended in the pediatric emergency department: First results in a tertiary hospital in Madrid. *Enfermedades infecciosas y microbiologia clinica (English ed)* 2024; **42**(7): 367-72.

79. Perramon-Malavez A, Hermosilla E, Coma E, et al. Effectiveness of Nirsevimab Immunoprophylaxis Against Respiratory Syncytial Virus-related Outcomes in Hospital Care Settings: A Seasonal Cohort Study of Infants in Catalonia (Spain). *Pediatr Infect Dis J* 2025.
